# Functional mutation, splice, distribution, and divergence analysis of impactful genes associated with heart failure and other cardiovascular diseases

**DOI:** 10.1038/s41598-023-44127-1

**Published:** 2023-10-05

**Authors:** Ishani Mhatre, Habiba Abdelhalim, William Degroat, Shreya Ashok, Bruce T. Liang, Zeeshan Ahmed

**Affiliations:** 1 Institute for Health, Health Care Policy and Aging Research, Rutgers University, 112 Paterson Street, New Brunswick, NJ 08901 USA; 2 Department of Genetics and Genome Sciences, UConn Health, 400 Farmington Ave, Farmington, CT USA; 3 Pat and Jim Calhoun Cardiology Center, UConn Health, 263 Farmington Ave, Farmington, CT USA; 4 UConn School of Medicine, University of Connecticut, 263 Farmington Ave, Farmington, CT USA; 5 Department of Medicine/Cardiovascular Disease and Hypertension, Robert Wood Johnson Medical School, Rutgers Biomedical and Health Sciences, 125 Paterson St, New Brunswick, NJ USA

**Keywords:** Cardiovascular diseases, Genomics

## Abstract

Cardiovascular disease (CVD) is caused by a multitude of complex and largely heritable conditions. Identifying key genes and understanding their susceptibility to CVD in the human genome can assist in early diagnosis and personalized treatment of the relevant patients. Heart failure (HF) is among those CVD phenotypes that has a high rate of mortality. In this study, we investigated genes primarily associated with HF and other CVDs. Achieving the goals of this study, we built a cohort of thirty-five consented patients, and sequenced their serum-based samples. We have generated and processed whole genome sequence (WGS) data, and performed functional mutation, splice, variant distribution, and divergence analysis to understand the relationships between each mutation type and its impact. Our variant and prevalence analysis found *FLNA*, *CST3*, *LGALS3*, and *HBA1* linked to many enrichment pathways. Functional mutation analysis uncovered *ACE*, *MME*, *LGALS3*, *NR3C2*, *PIK3C2A*, *CALD1*, *TEK*, and *TRPV1* to be notable and potentially significant genes. We discovered intron, 5ʹ Flank, 3ʹ UTR, and 3ʹ Flank mutations to be the most common among HF and other CVD genes. Missense mutations were less common among HF and other CVD genes but had more of a functional impact. We reported *HBA1*, *FADD*, *NPPC*, *ADRB2*, *ADBR1*, *MYH6*, and *PLN* to be consequential based on our divergence analysis.

## Introduction

Cardiovascular disease (CVD) is the leading cause of death and mortality internationally, with as many as 655,000 deaths per-year^1,2^. In 2015, there were approximately 422.7 million cases of CVD and 17.92 million deaths reported^3^. CVD include primary pathologies such as heart failure (HF), cardiac arrhythmias, venous thromboembolism, cerebrovascular and peripheral arterial disease, coronary heart disease (CHD), coronary artery disease (CAD), and atheromatous vascular disease (AVD)^4,5^. The most common causes of CVD mortality include but are not limited to ischemic and nonischemic HF and stroke^3^. Hence, one of the focuses of life science involves investigating genetic epidemiology of CVD. Due to the complex nature, risk factors, inherent genetic makeup, and progression of CVD, personalized treatment is believed to be essential^6^. Precision medicine involves integrating clinical and multi-omics/genomics data for predictive and personalized medicine within a diverse CVD population^7^. It focuses on analyzing genetic composition of patients to identify the key biomarkers and increase understanding of the pathophysiology of CVD^8^.

CVD is a complex, partially heritable condition, encompassing a range of conditions from CHD to myocardial infarction^9^. By utilizing high-quality sequenced DNA of transcribed genes, we can be better informed of a CVD patient’s inherent genetic makeup and factors that may contribute to increased susceptibility for CVD^10^. Whole-Genome-Sequencing (WGS) has been proven to be one of the most recommended techniques to sequence DNA and capture all genetic variations. Various WGS based studies have focused on investigating mutated genes with altered expression^11–13^, and discovered underlying genetic etiology in CVD patients^14,15^. State of the art studies have supported the claim that performing variant analysis will assist in understanding of the complex pathophysiology of CVD progression through the application of multiple biomarkers^16–18^. However, we are still in the early stages of developing a comprehensive database of genetic biomarkers for CVD to assist in predictive analysis and deep phenotyping^19–22^. Previously, we have explored and discussed diverse genomic strategies that investigate genes linked to AF, HF, and other CVDs^23^. In this study, we aimed to investigate genes primarily associated with HF and other CVDs by analyzing genetic variants that correlate with CVD phenotype^24^.

## Material and methods

**Figure 1:**
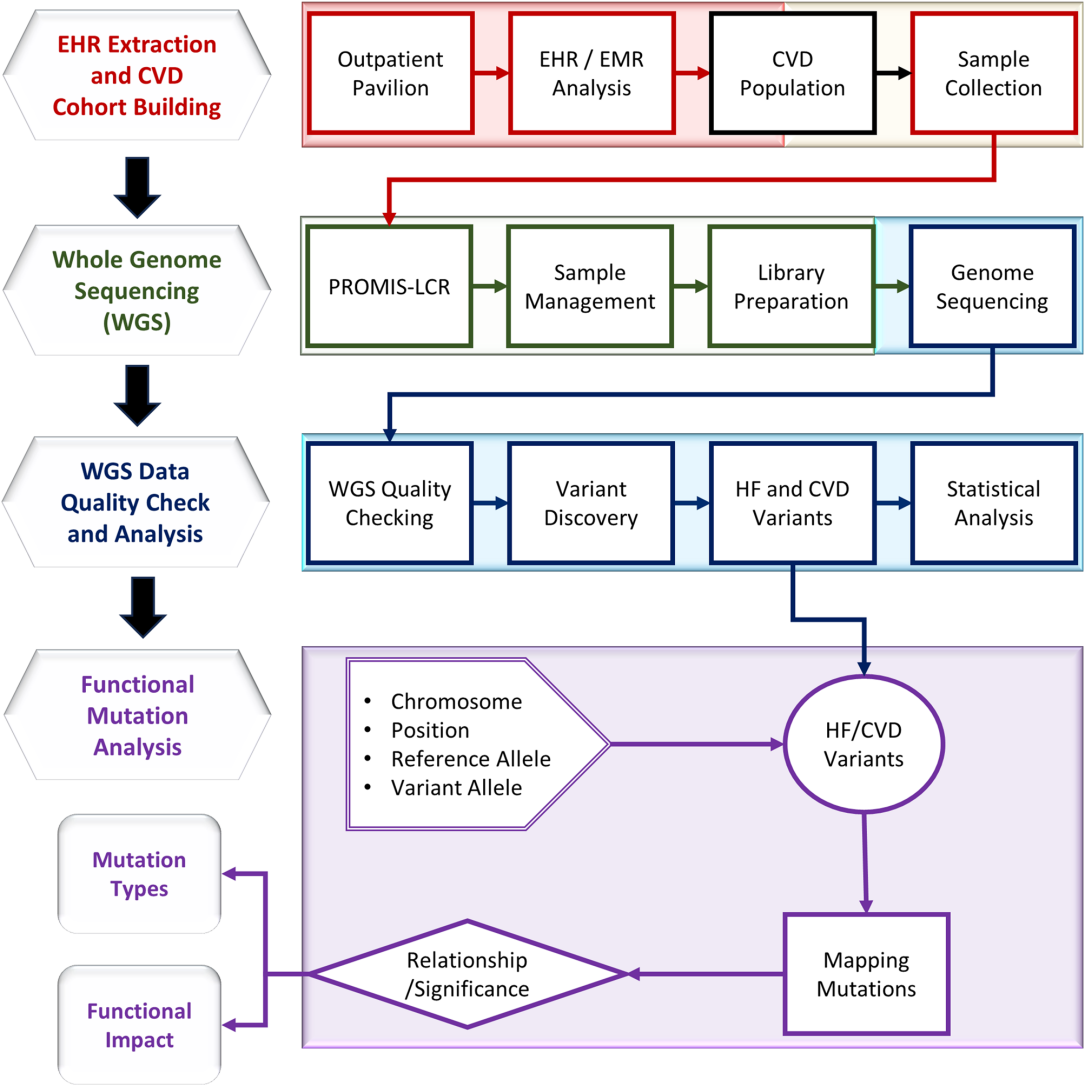
Study design. Overall research methodology includes four major steps: (1) EHR extraction and CVD cohort building; (2) Whole Genome Sequencing (WGS); (3) WGS data quality check and analysis; and (4) Functional mutation analysis to identify mutation types and their functional impact. A more detailed overview of our methodology is highlighted in the right panel. Different approaches grouped within the same major steps utilize identical colors.

Achieving the goals of this study, we analyzed electronic health records (EHR) received from EPIC health system to build a cohort of thirty-five patients with CVD (Fig. 1). Our selection criteria mainly included adult and aging CVD patients with HF phenotype. In addition, we collected information centered on their age, gender, ethnicity, medical details, and demographics. We identified 21 male and 14 female individuals (60% male and 40% female population) aged between 24 and 94 years (details are attached in supplementary material S6). These patients were clinically diagnosed with CVD and CMS/HCC HF, as well as cardiomyopathy, hypertension, obesity, type 2 diabetes mellitus, asthma, high cholesterol, hernia, chronic kidney, joint pain, myalgia, dizziness and giddiness, osteopenia of multiple sites, chest pain, and osteoarthritis. We collected blood samples from these CVD patients and extracted DNA. We have utilized our in-house developed applications to support patient consenting, sample collection, data management, and EHR extraction, transfer, loading (ETL) and analysis^25,26^. Written informed consent was obtained from all subjects. All procedures performed in studies involving human participants were in accordance with the ethical standards of the institution and with the 1964 Helsinki declaration and its later amendments or comparable ethical standards. All human samples were used in accordance with relevant guidelines and regulations, and all experimental protocols were approved by the Institutional Review Board (IRB) at UConn Health.

We performed high-throughput WGS of collected blood samples, and processed sequence data for quality checking (QC) and variant discovery (QC report is attached in supplementary material S7). We utilized our in-house built pipeline (JWES) for WGS data processing, management, visualization (Circos plots), and gene-variant discovery, annotation, prediction, and genotyping^27^. JWES mainly utilizes the Burrows-Wheeler Aligner (BWA, version 0.7.17) for mapping sequence data against the reference human genome^28^, and Genome Analysis Toolkit (GATK, version 3.8) for the variant discovery^29^. We performed variant calling of the whole genome using JWES for all subjects but focused on targeted HF and other CVD genes for further analyses. Utilizing significant results of differentially regulated genes from our previous expression and enrichment analysis^30^ that were validated through our gene-disease-variant database^31^, we generated a list of forty-one HF and twenty-three other CVD genes (Supplementary material S1). We calculated pLI scores for these genes using The Genome Aggregation Database (gnomAD) to better contextualize these mutation’s effects on disease (Supplementary Tables S8, S9)^32^.

We conducted functional mutation, splice, variant distribution, and divergence analysis to understand the relationships between each mutation type and its impact. We utilized Scale-Invariant Feature Transform (SIFT)^33–35^, Polymorphism Phenotyping v2 (PolyPhen-2)^36^, and MutationAssessor^37^ to classify the biological and functional impacts of the variant data. SIFT supported in analyzing the impact of coding variants on the function of protein and identify variants that have a causal relationship to the manifestation of HF and other CVDs^34^. PolyPhen-2 garnered a wide breadth of information about the substitution site of the coding variant and identified the specific gene sequences and structural features of the substitution site. It analyzed single-nucleotide polymorphism (SNP) substitutions and predicted the functional impact of the mutations. Then, MutationAssessor differentiated between specificity scores to account for functional shifts between subfamilies, proteins, and conserved patterns^38,39^. Scores from SIFT, PolyPhen-2, and MutationAssessor are included in our supplementary material S1.

We preformed splice mutation analysis and a Jensen-Shannon Divergence (JSD)-based Method (JS-MA) for the measurement and variant distribution analysis^40^. We reported our findings on RNA, silent, 3ʹ UTR, 3ʹ Flank, 5ʹ UTR, 5ʹ Flank, intron, truncating, splice, and missense mutations for genes associated with HF and other CVDs. We analyzed RNA, truncating, missense, 3ʹ UTR, and 5ʹ UTR mutations to study the structural consequences of the cellular proteome. These mutations affect the functionality of the protein produced and can lead to a gain or loss of function^41–45^. Mutations in RNA can lead to changes in the sequence of nucleotides, which can affect the structure and function of the RNA molecule and subsequently impact molecular processes^41^. RNA-based mutations include but are not limited to point, nonsense, silent and missense^41^. We observed the suppression or overexpression of a gene by investigating 3ʹ Flank and 5ʹ Flank mutations^43^. By examining intro and splice mutations, we gained a better understanding of the effect that they can have on RNA splicing process resulting in a decrease efficiency of mRNA translation^46–48^.

Utilizing JS-MA, we conducted a genome-wide search for complex gene-disease interactions, helping us better understand the effects that gene mutations can have on a phenotypic state^40^. Divergence analysis involved comparing each gene’s distribution of mutations to a weighted average of all genes in that disease type. Variance from this distribution indicates an overrepresented mutation type among HF and CVD patients. We calculated Jensen-Shannon Divergence (JSD) scores to evaluate the similarity between the two distributions. The JSD score measured the variance associated with two distributions and provided a statistical quantification on the influence of specific mutations on disease types^40^. A JSD score closer to ‘1’ indicates the highest variance denoting a unique mutation profile with greater impact. We identified notable and potentially significant genes based on whether the HF and other CVD genes met a certain threshold using their calculated JSD scores. We compared proportion distributions of unique genes and a weighted average distribution of all genes within the disease type. To ensure the validity of our results, we tried to account for confounding variables and found that the biological variables such as age of onset of HF, severity of disease, alcoholic cardiomyopathy and different aetiologias can be ruled out as they did not have any significant impact on the outcome of our study^49–51^.

### Ethical approval and consent to participate

Informed consent was obtained from all subjects. All human samples were used in accordance with relevant guidelines and regulations, and all experimental protocols were approved by the Institutional Review Board.

## Results

**Figure 2:**
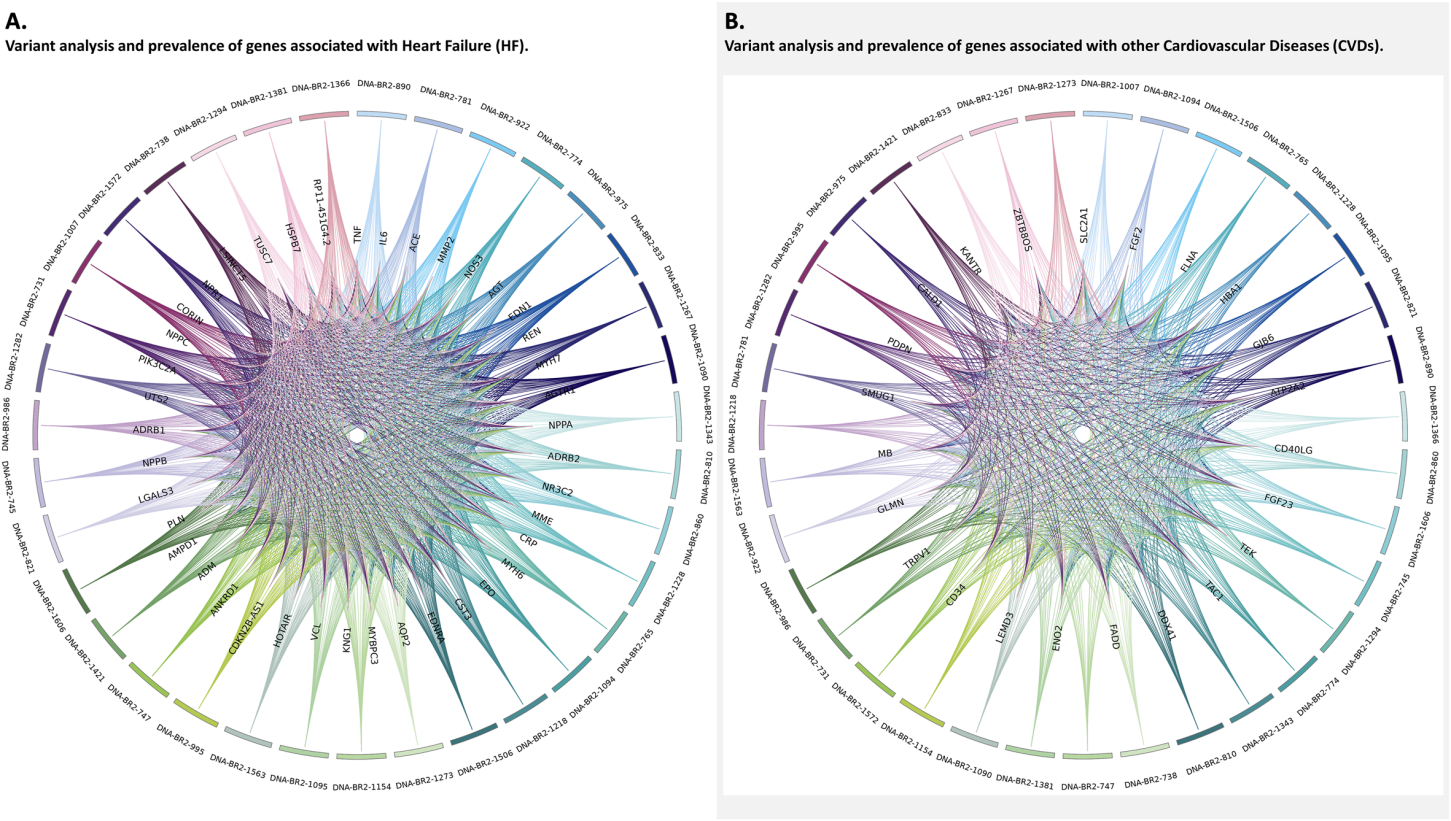
Gene-variant analysis of significant protein-coding genes associated with heart failure (HF) and other cardiovascular diseases (CVD) discovered through mutation analysis. (**A**) Variant analysis and prevalence of significant HF genes. (**B**) Variant analysis and prevalence of significant other CVD genes. These Circos plots are produced using the visualization module of the JWES pipeline^26^.

**Figure 3:**
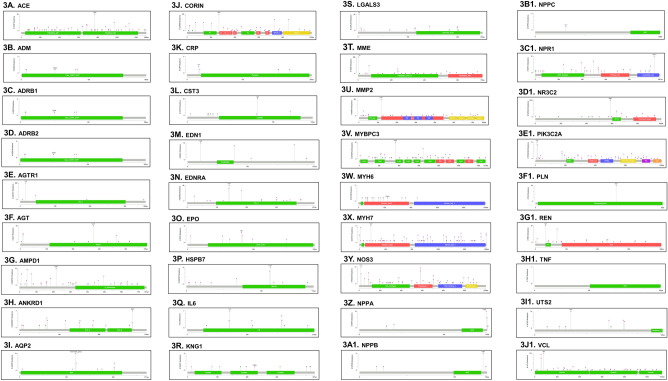
Functional mutation analysis of heart failure (HF) genes. Lollipop graphs of *ACE, ADM, ADRB1, ADRB2, AGTR1, AGT, AMPD1, ANKRD1, AQP2, CORIN, CRP, CST3, EDN1, EDNRA, EPO, HSPB7, IL6, KNG1, LGALS3, MME, MMP2, MYBPC3, MYH6, MYH7, NOS3, NPPA, NPPB, NPPC, NPR1, NR3C2, PIK3C2A, PLN, REN, TNF, UTS2*, and *VCL*. Green represents Missense mutations; black represents Truncating mutations; brown represents Inframe mutations; and purple represents Fusion mutations.

**Figure 4:**
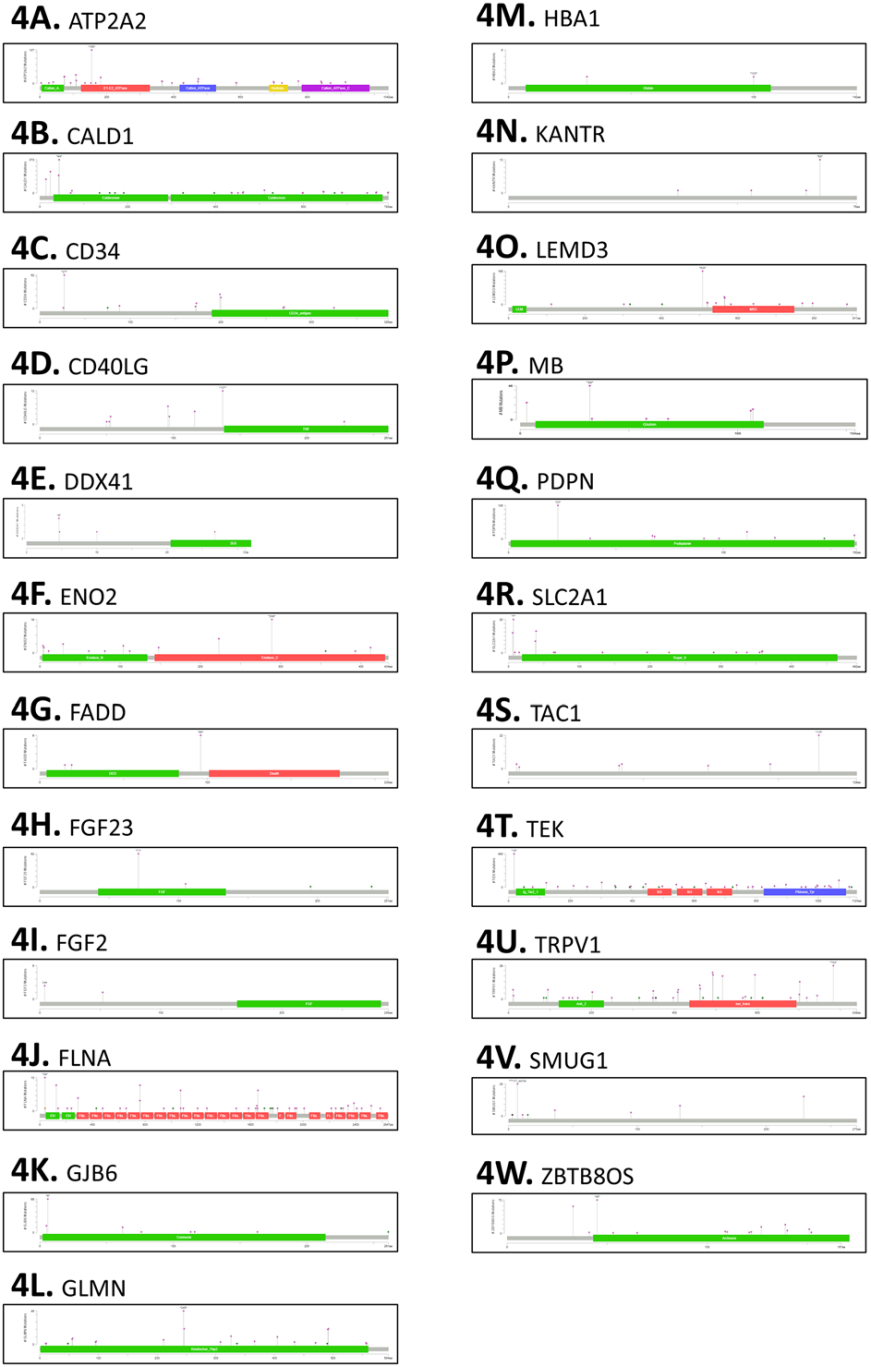
Functional mutation analysis of other cardiovascular disease (CVDs) genes. Lollipop graphs of: *ATP2A2, CALD1, CD34, CD40LG, DDX41, ENO2, FADD, FGF23, FGF2, FLNA, GJB6, GLMN, HBA1, KANTR, LEMD3, MB, PDPN, SLC2A1, SMUG1, TAC1, TEK, TRPV1*, and *ZBTB8OS*. Green represents Missense mutations; black represents truncating mutations; brown represents inframe mutations; and purple represents fusion mutations.

Our variant analysis started with examining the variant distribution and prevalence of HF and CVD genes to better understand the frequency of these genetic variants. We generated Circos plots and observed a total of 229,963 variants for HF genes (Fig. 2A). For CVD genes, we visualized a total of 389,761 variants (Fig. 2B). The outer circle of the plot represents patient sample IDs, while the inner circle represents genes. Figure 2A has more HF genes along the inner circle compared to Fig. 2B which has fewer other CVD genes. Next, we conducted functional mutation analysis to evaluate the effects of disease-causing alleles for HF and other CVDs. We detected consistent distribution of mutation types for the mapped genes. These mutations included Missense, Splice, Truncating, Intron, 5' Flank, 5' UTR, 3' Flank, 3' UTR, Silent, and RNA-driven mutations for HF (Table 1) and other CVDs (Table 2). We generated lollipop plots for HF and other CVD genes to visualize the functional impact for each mutation type (Figs. 3 and 4). Currently, there are 373 datasets and a total of 162,055 mutations referenced in cBioPortal. These datasets referenced do not encompass all variants that we reported in our prevalence analysis. Due to this limitation, some genes were not annotated and visualized. These genes include *CDKN2B-AS1*, *HOTAIR*, *LSINCT5*, *RP11-451G4.2*, and *TUSC7*. Missense mutations had higher functional impacts and were more likely to be ‘possibly or probably damaging.’ We measured the effect of mutations using a score assigned to predict whether an amino acid substitution affects protein function. SIFT scores varied from 0.0 to 1.0. Mutations ranging from 0.0 to 0.5 were considered “deleterious” while those ranging from 0.5 to 1.0 were “tolerated/benign.” Additionally, scores regarded as "deleterious low confidence" were less likely to have a phenotypic effect than "deleterious" while "tolerated low confidence" were more likely to have a phenotypic effect than 'tolerated'^35^.

**Table 1:** Functional mutation analysis of genes associated with heart failure disease.

Gene Name	Missense	Splice	Truncating	Intron	5ʹ Flank	5ʹ UTR	3ʹ Flank	3ʹ UTR	Silent	RNA	Total	Failed Mutations
ACE	12	4	0	120	24	N/A	66	6	15	N/A	247	60
ADM	1	0	0	3	28	N/A	16	2	1	N/A	51	29
ADRB1	5	0	0	N/A	30	2	31	8	5	N/A	81	1779
ADRB2	5	0	0	N/A	38	3	29	2	4	N/A	81	1866
AGTR1	0	0	0	257	40	1	31	12	2	N/A	343	479
AGT	3	0	0	67	37	N/A	24	2	2	N/A	135	718
AMPD1	4	1	1	103	9	N/A	N/A	N/A	1	N/A	119	78
ANKRD1	3	0	0	31	15	1	29	2		N/A	81	61
AQP2	0	1	0	28	25	1	8	17	2	N/A	82	85
CDKN2B-AS1	X	X	X	X	X	X	X	X	X	X	X	X
CORIN	5	2	0	1234	17	N/A	2	9	6	N/A	1275	404
CRP	1	0	0	2	32	N/A	8	5	2	N/A	50	291
CST3	2	0	0	16	33	2	67	6	2	N/A	128	254
EDN1	1	0	0	33	32	N/A	38	3	1	N/A	108	1320
EDNRA	0	0	0	296	34	3	21	8	2	N/A	364	438
EPO	0	0	0	9	25	3	15	4	N/A	N/A	56	124
HOTAIR	X	X	X	X	X	X	X	X	X	X	X	X
HSPB7	1	0	0	19	66	3	46	19	4	N/A	158	14
IL6	2	0	0	22	15	N/A	31		1	N/A	71	95
KNG1	3	1	1	15	N/A	2	2	1	4	N/A	29	307
LGALS3	4	1	0	83	61	1	N/A	N/A	N/A	N/A	150	474
LSINCT5	X	X	X	X	X	X	X	X	X	X	X	X
MME	3	3	0	953	63	1	1	18	2	N/A	1044	1096
MMP2	1	1	0	456	68	2	N/A	9	9	N/A	546	741
MYBPC3	9	1	1	72	11	N/A	N/A	N/A	10	N/A	104	84
MYH6	3	1	0	74	N/A	N/A	N/A	N/A	11	N/A	89	51
MYH7	4	5	0	105	35	N/A	29	1	16	N/A	195	231
NOS3	7	0	0	143	39	1	1	2	6	N/A	199	116
NPPA	3	0	1	16	25	N/A	N/A	4	N/A	N/A	49	52
NPPB	2	0	0	5	28	N/A	40	N/A	1	N/A	76	244
NPPC	0	0	0	1	23	N/A	36	N/A	N/A	N/A	60	457
NPR1	5	0	0	53	13	3	37	N/A	1	N/A	112	151
NR3C2	3	2	0	2057	27	1	2	6	5	N/A	2103	1079
PIK3C2A	1	1	0	423	37	2	29	4	5	1	503	326
PLN	0	0	0	49	N/A	N/A	N/A	9	N/A	N/A	58	46
REN	1	1	0	108	34	3	N/A	N/A	2	N/A	149	150
RP11-451G4.2	X	X	X	X	X	X	X	X	X	X	X	X
TNF	0	0	0	7	15	N/A	N/A	2	N/A	N/A	24	780
TUSC7	X	X	X	X	X	X	X	X	X	X	X	X
UTS2	3	0	0	30	37	N/A	19	N/A	1	N/A	90	806
VCL	2	0	0	522	42	1	N/A	5	7	N/A	579	875

Tabulated information includes, gene name, missense, splice, truncating, intron, 5ʹ flank, 5ʹ UTR, 3ʹ flank, 3ʹ UTR, silent, RNA, total and failed mutations.

**Table 2:** Functional mutation analysis of genes associated with other cardiovascular diseases.

Gene name	Missense	Splice	Truncating	Intron	5ʹ UTR	5ʹ Flank	3ʹ UTR	3ʹ Flank	Silent	RNA	Total	Failed Mutation
ATP2A2	0	2	0	270	6	11	14	24	3	0	330	478
CALD1	8	0	0	1100	1	66	7	26	2	0	1210	492
CD34	1	1	0	109	N/A	30	39	1	1	0	182	701
CD40LG	0	0	0	32	N/A	18	9	N/A	2	0	61	56
DDX41	0	2	0	17	N/A	4	2	N/A	1	0	26	13
ENO2	1	1	0	51	1	N/A	8	N/A	1	0	63	79
FADD	0	0	0	9	2	36	3	20	N/A	0	70	706
FGF23	2	0	0	68	N/A	33	5	17	N/A	0	125	451
FGF2	0	0	0	394	N/A	37	N/A	N/A	3	0	434	694
FLNA	2	6	0	82	N/A	N/A	2	10	11	0	113	73
GJB6	1	0	0	88	3	42	4	36	1	0	175	2133
GLMN	3	1	0	172	N/A	1	1	4	N/A	0	182	96
HBA1	0	0	0	2	N/A	12	1	15	N/A	0	30	28
KANTR	0	0	0	145	4	17	7	20	1	3	197	165
LEMD3	2	3	0	271	N/A	19	4	19	4	0	322	525
MB	0	0	0	95	1	39	4	49	2	0	190	738
PDPN	1	0	0	203	1	35	20	1	2	0	263	351
SLC2A1	0	1	0	216	2	N/A	5	30	7	0	261	230
TAC1	0	0	0	36	N/A	24	5	38	N/A	0	103	2518
TEK	8	1	0	1120	N/A	46	8	69	9	0	1261	518
TRPV1	8	0	1	207	3	68	7	38	5	0	337	256
SMUG1	1	1	1	61	1	17	4	26	N/A	0	112	433
ZBTB8OS	0	0	0	197	N/A	N/A	12	23	1	0	233	205

Tabulated information includes, gene name, missense, splice, truncating, intron, 5ʹ flank, 5ʹ UTR, 3ʹ flank, 3ʹ UTR, silent, RNA, total and failed mutations.

Functional impact scores from PolyPhen-2 ranged from 0.0 to 1.0 with values closer to 1.0 being ‘possibly or probably damaging’ and those closer to 0 being ‘benign^36^.’ *The AGTR1, AQP2, EDNRA, EPO, NPPC, PLN*, and *TNF* genes had no missense mutations and provided no further information regarding functional impact for the mutations. *ACE* had the highest number of missense mutations: twelve mutations in total. Five of those missense mutations were found to have some negative impact on the function of the protein. *NR3C2* had the highest number with a total of 2,057 intron mutations. *PIK3C2A* was the only gene with an RNA-based mutation. Aside from the RNA mutation, the rarest mutation type was truncating mutations. *AMPD1*, *KNG1*, *MYBPC3*, and *NPPA* were found to have a truncating mutation. Splice and 5ʹ UTR were also found to be less common. Genes such as *CORIN*, *MMP2*, *MYBPC3*, *NOS3*, and *PIK3C2A* had more specific functional protein domains (Pfam domains), on average, compared to the other HF genes. From the genes investigated in our study, we found the *ACE, MME*, *LGALS3*, *NR3C2*, and *PIK3C2A* genes to be more significant based on various criteria such as the largest number of mutations mapped, rare mutation types, and highest number of mutations with functional impact. Previous literature has already linked or hypothesized *ACE, MME, LGALS3, NR3C2*, and *PIK3C2A* to be significant genes and potential biomarkers for CVDs^52–56^. Further research must be conducted to solidify these claims and increase confidence regarding the significance of these genes. We reported different types of mutations and their impact on all HF genes in Supplementary material S2.

For other CVD genes, *CALD1, TEK, TRPV1, ATP2A2*, and *SMUG1* were discovered to be more significant based on the same criteria which includes genes with the highest number of mutations mapped, rare mutation types, and the largest number of mutations with functional impact. *CALD1*, *TEK*, and *TRPV1* all had the highest number of missense mutations, with eight missense mutations each. In the *CALD1* gene, the breakdown was one tolerated low confidence and benign, two deleterious and benign, two deleterious low confidence and benign, one deleterious and possibly damaging, one tolerated and benign, and one deleterious and probably damaging; hence, six of the eight mutations had some negative functional impact on the protein. In the *TEK* gene, the breakdown was five tolerated and benign, one tolerated and probably damaging, one deleterious and possibly damaging, and one deleterious and benign; hence, three of the eight mutations had some negative functional impact on the protein. In the *TRPV1* gene, the breakdown was seven tolerated and benign and one deleterious and benign; hence, only one of the eight mutations had some negative functional impact on the protein. *CALD1, TEK*, and *TRPV1* were found to be the most significant of the investigated genes as they have the largest number of functional mutations. *CALD1* and *TEK* also had the highest number of mutations mapped in total. Other CVD genes mutations including *ATP2A2* and *SMUG1* were discovered to have rare mutation types. We reported no missense mutations for multiple genes, therefore no further information regarding functional impact scores could be found. These genes included *ATP2A2*, *CD34*, *CD40LG*, *DDX41*, *FADD*, *FGF2*, *FLNA*, *HBA1*, *KANTR*, *MB*, *SLC2A1*, *TAC1*, and *ZBTB8OS*. Previous literature has linked *CALD1, TEK, TRPV1, ATP2A2*, and *SMUG1* to CVDs, supporting the findings from our functional mutation analysis^57–61^. Further research must be conducted to solidify these claims regarding the significance of these genes. We reported different types of mutations and their impact on all CVD genes in Supplementary material S3.

**Figure 5:**
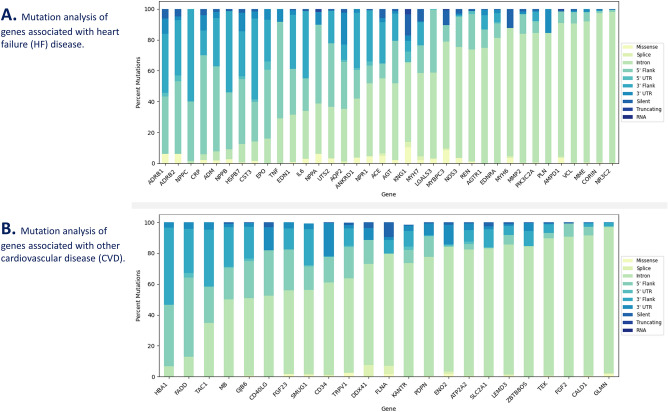
Splice mutation analysis of genes associated with heart failure (HF) and other cardiovascular disease (CVDs). Figure reports findings on missense, splice, truncating, intron, 5ʹ flank, 5ʹ UTR, 3ʹ flank, 3ʹ UTR, silent and RNA mutations for genes associated with HF (**A**) and other CVDs (**B**).

Next, our splice mutation analysis uncovered mutation frequencies for the list of significant mutated genes generated after performing high-throughput WGS and utilizing JWES for WGS data processing and gene-variant discovery^27^. We were able to analyze the percentages of each mutation (missense, splice, truncating, intron, 5ʹ flank, 5ʹ UTR, 3ʹ flank, 3ʹ UTR, silent and RNA) in comparison to each other (Fig. 5). We reported that intron, 5ʹ Flank and 3ʹ Flank mutations were present in high frequencies in genes associated with HF (Fig. 5A) and other CVDs (Fig. 5B). *NR3C2* had the highest number of intron mutations with a total of 2,057. *PIK3C2A* was the only gene with an RNA-based mutation. Aside from the RNA mutation, the rarest mutation type was truncating mutations. *AMPD1*, *KNG1*, *MYBPC3*, and *NPPA* were found to have a truncating mutation. Splice and 5ʹ UTR were also less common or rarer mutation types (Fig. 5A). Among the genes associated with other CVDs*, TEK* had the highest number of intron mutations, with a total of 1,120. RNA mutations were the rarest in CVD genes as well, with *KANTR* being the only gene possessing RNA mutations. Truncating mutations were also very rare. *TRPV1* and *SMUG1* possessed truncating mutations (Fig. 5B).

**Figure 6:**
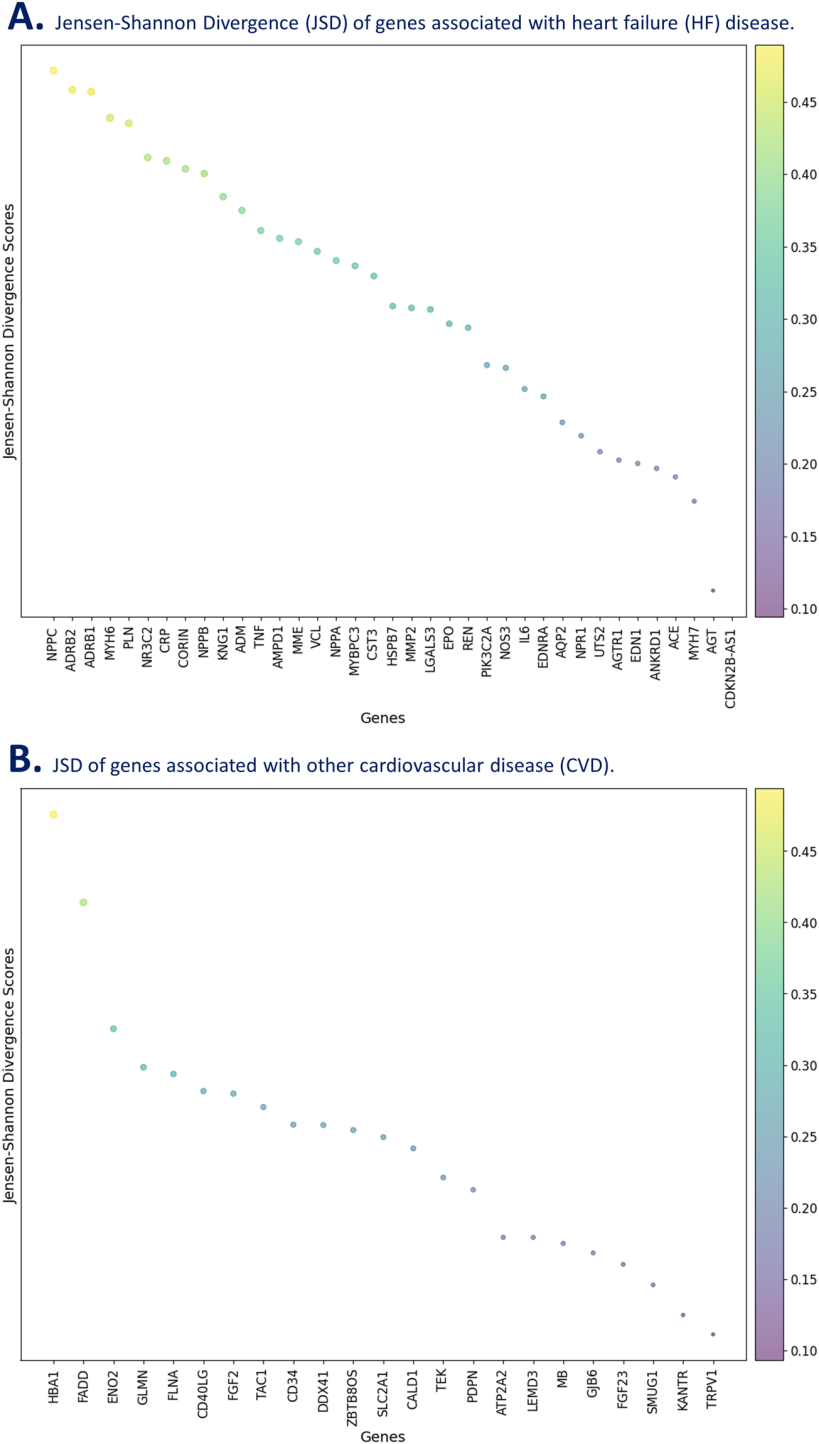
Jensen-Shannon divergence-based (JSD) statistical analysis and variant distribution analysis of genes associated with heart failure (HF) and other cardiovascular diseases (CVDs). Figure reports JSD scores of genes associated with HF (**A**) and other CVDs (**B**).

We implemented JS-MA and the computed JSD scores highlighted the variance for all genes in relation to the disease (HF or other CVDs). The JSD scores for both HF and other CVD genes ranged from 0.09 to 0.49 with the diameter of each circle representing the score (Fig. 6). For the genes associated with HF, we observed five genes to be highly variant compared to others. These included *NPPC*, *ADRB2*, *ADRB1*, *MYH6* and *PLN* with JSD scores of 0.489, 0.474, 0.473, 0.453, and 0.449 respectively. *NR3C2*, *CRP*, *CORIN*, *NPPB*, *KNG1*, and *ADM* had moderate JSD for HF (Fig. 6A). For genes associated with other CVDs, we identified one gene, *HBA1*, to be extremely significant with a JSD of 0.493. We found *FADD* to have the second highest variance with a score of 0.425. Other genes with moderate JSD included *ENO2*, *GLMN*, *FLNA*, *CD40LG, FGF2*, *TAC1*, *CD34*, *DDX41*, *ZBTB8OS*, *SLC2A1*, *CALD1*, *TEK*, and *PDPN* (Fig. 6B). We found the following genes to have the highest variance: *HBA1*, *FADD*, *NPPC*, *ADRB2*, *ADRB1*, *MYH6*, and *PLN*. The exact JSD scores for all genes can be found in Supplementary material S1. Processed variant data of genes associated with HF and other CVDs are attached in the supplementary material (S4, S5, and S10).

We utilized a variety of analyses to identify notable genes including variant and prevalent analysis, functional mutation analysis, splice, and divergence analysis. Next, we performed comparative analysis to identify which genes were found to be notable and potentially significant in more than one method of analyses. The *HBA1* gene had a high JSD score and was observed in multiple enrichment pathways using our variant analysis and prevalence analysis. Hemoglobin subunit alpha 1 is involved in controlling pathways such as oxygen-carbon dioxide exchange in erythrocytes as well as cellular response to stimuli^62^. Mutations in *HBA1* have been found to be associated with multiple CVDs including but not limited to CAD^62^. Loss of function in *HBA1* can lead to Hemoglobin H disease, more commonly known as Alpha-thalassemia^62^. We found *LGALS3* reported in our variant as well as functional mutation analysis. *LGALS3* codes for Galectin-3 (Gal-3), a protein that plays an important role in cell proliferation, adhesion, differentiation, and apoptosis. Recent studies have linked Gal-3 levels to organ health and increase in Gal-3 levels have been associated with fibrotic and inflammatory diseases^63^. *CALD1* and *TEK* were found to be highly significant based on our functional mutation analysis and had moderate JSD scores. *CALD1* is a protein coding gene that affects myosin in the smooth muscle. Mutations in *CALD1* have been associated with CVDs including but not limited to cardiomyopathy^64^. *TEK* is involved in many biological pathways such as influencing the growth of blood vessels. Mutations in this gene can lead to abnormal formation of blood vessels and the heart^65^. From the HF and other CVD genes, *HBA1*, *LGALS3,* and *TEK* had the strongest evidence of being significant and linking to CVDs based on the multiple analyses conducted as well as previous literature.

Comparing the results between HF and other CVD genes, we discovered many trends and distribution of mutation types and variations to be similar for both HF and other CVD genes (Fig. 2A,B). Most lollipop plots for HF and other CVDs had only one type of Pfam domain mapped for the corresponding gene (Figs. 3, 4). For HF genes, eleven genes in total (*CORIN, MME, MMP2, MYBPC3, MYH6, MYH7, NOS3, NPR1, NR3C2, PIK3C2A,* and *REN*) had two or more Pfam domains mapped (Fig. 3). For other CVD genes, the following seven genes were discovered to have two or more Pfam domains: *ATP2A2, LEMD3, ENO2, FADD, TEK, TRPv1, FLNA* (Fig. 4). HF genes, on average, had more Pfam domains that were able to be mapped. The most common mutation type for both HF and other CVDs was intron mutations with the least common being RNA, silent, and truncating mutation types. One major difference was that HF genes had an overall greater number of mutation types including RNA and truncating, both of which were not found in the other CVD genes (Fig. 5A,B). Understanding the common trends and variations in mutation distributions for HF and other CVDs can reveal similarities between the pathophysiology of multiple diseases and highlight the importance of further research to understand the relationship between HF and other CVD genes.

## Discussion

*LGALS3* codes for Gal-3 and recent studies have linked Gal-3 levels to organ health as well as fibrotic and inflammatory diseases^63^. *LGALS3* had four missense mutations; the mutation mapped to P64H had a high functional impact (deleterious and probably damaging were SIFT and PolyPhen-2 scores), and the other two missense mutations were mapped to T98P and R183K; both mutations had low functional impact. Our analysis suggests *LGALS3* could also be linked to CVDs in addition to fibrotic diseases. Further studies are needed to confirm this relationship. A previous trial linked *MME* with CVDs and found HF patients had less chances of being hospitalized if treated with an angiotensin receptor neprilysin inhibitor^66^. Although the remaining genes (*CST3, NR3C2, PIK32CA, TNF*, and *VCL*) had low functional impact for mutations, *PIK32CA* was also significant since it was the only gene out of thirty-six HF genes that produced a lollipop graph with an RNA mutation type. Additionally, we found *NPPC, ADRB2, ADBR1, MYH6* and *PLN* genes to have high variance based on JS-MA.

When conducting mutation analysis, our study was able to generate functional mutation scores for *LEMD3* and *SMUG1*; for the other genes, no functional mutation information could be found, as there were no missense mutations present. *LEMD3* had one mutation mapped with a high functional impact (deleterious and possibly damaging for the SIFT and PolyPhen-2 scores) and one mutation with low functional impact. Mutations in *LEMD3* have been linked to various conditions such as Buschke–Ollendorff^67^ and our study suggests the gene can have further links to CVDs. Less gene expression of *SMUG1* has been linked to breast cancer^68^. *SMUG1* had one mutation with low functional impact, which suggests further research should be conducted to assess its association with CVDs as well. We found *HBA1* and *FADD* were found to be extremely significant using JS-MA. Mutations in *HBA1* have been found to be associated with multiple CVDs including but not limited to CAD^64^. While mutations in *FADD* have been associated with post-ischemic HF, further studies are needed to study if *FADD* can be used in gene therapy for HF treatment^65^. Further research is needed for *LEMD3, SMUG1, HBA1, FLNA, ZBTB8OS, and SLC2A1* since they were found significant in multiple analyses conducted.

Additional genes from our variant and functional mutation analysis were reported to be significant. From the HF genes, *ACE* was found to have the largest number of missense mutations with a high functional impact; in the CVD genes, *CALD1*, *TEK*, and *TRPV1* genes had the largest number of mutations with high functional impact. Future studies are needed to be better informed and targeted towards certain genes for mutation analysis and disease-specific variants. Findings from our functional mutation analysis warrant further study of the gene-disease causal relationships involving HF and CVD genes, especially *ACE, CALD1, TEK*, and *TRPV1*. Significant genes noted in our current study were also supported by findings from our previous RNA-seq driven gene differential expression and pathway enrichment analysis. Genes such as *FADD*, *HBA1* and *LGALS3* were found to be differentially expressed in HF patients^30^. While *CALD1*, *TEK*, and *TRPV1* showed low expression in HF patients compared to healthy controls^30^. Most of our biological findings for significant genes are thus validated by previous gene-disease annotation, phenotyping as well as mRNA abundance analysis^30^. We found *ADRB1, ADRB2,* and *NPPC* to have great variance and significance based on JS-MA from our previous variant analysis from a separate ensemble of CVD patients^69^. Thus, supporting our claim that these genes have significant or altered expression in CVD patients. Additionally, we observed that *ACE* and *CALD1* were highly associated with CVDs and played a major role in disease prediction based on our Artificial Intelligence (AI) and Machine Learning (ML) driven analysis^70^.

There were some limitations to using the cBioPortal Mutation Mapper. The total amount of mutations discovered by our previous study for each significant HF and other CVD gene were not all able to be mapped onto the lollipop graphs^26,27^. There were a significant number of mutations that failed to be annotated due to insufficient information in the reference database. Results showed that seven HF genes studied possessed mutations whose functional impacts could not be tracked due limitations of the software; the same was true for thirteen CVD genes. The cBioPortal software was unable to support this information since the mutations discovered were novel and the database has not been updated yet. These limitations prevented a complete lollipop plot of mutation distributions from being generated for each HF and CVD gene. However, based on the numerous mutations that were mapped, significant patterns were discovered. Another limitation of our study was the sample size utilized that can limit the generalization of our findings. To partially address this limitation, we have conducted an additional whole genome and variant analysis on an alternative group of consented CVD patients to support and validate our findings^53^. Additionally, we plan on expanding our cohort in the future to include diverse individuals based on race, ethnicity, and socioeconomic factors to better highlight the importance and frequency of mutations linked to frequently studied HF and CVD genes.

Our methodology involved using JWES for WGS data processing and utilizing GATK for the identification of point mutations. Moving forward, the inclusion of other variation types including copy number variations (CNV), structural variants (SV), and short tandem repeats (STR) may increase or decrease the significance of genes depending on a variety of factors. Unlike SNPs which are variations of single nucleotide in a specific genome location, STRs are variations of the number of repeating DNA sequences. A previous study found that SNPs are considered a viable replacement for STRs to detect the structure of a population^71^. SVs are defined as a DNA region of about one kilobase (kb) and can include inversions or insertions and deletions, also known as CNVs^72^. While SNPs affect splicing or transcription and are present in coding or non-coding regions, CNVs are defined as sequence variants that can be as large as several megabases (Mbs) in size. CNVs have been linked to the pathogenesis of complex diseases; studies reveal that when associations exist between CNVs and SNPs, the coexistence frequency, and the type of CNV can lead to an overestimation or underestimation of the gene significance. The application of a joint analysis of CNVs and SNPs may address these current limitations and provide more accuracy in identifying significant genes moving forward^70^.

To study chronic diseases such as CVDs with complex pathophysiology, conducting multiple analyses with over-compassing methodologies is essential. The overall goal of the study was to conduct a combination of variant distribution and prevalence, functional mutation, splice mutation and divergence analysis to identify the significant impact of these mutations on the pathology of CVDs. Our results reinforce the established relationship between significant genes highlighted in previous literature and their impact on CVDs. Further research can be conducted to validate our claims regarding potentially significant genes by widening the sample size of consented patients to estimate trends within a population. This is a goal we hope to accomplish in the future. It is of paramount importance to fully understand the genetic basis of diseases, especially common ones, distinguish the genes which predispose an individual to medical conditions, and how rare genetic variations play a role in disease manifestation^74^. Further inquiry into these genes may foster the development of novel clinical tools that will improve personalized medical treatment for HF and other CVD patients. Once the individual’s genetic makeup is considered, medical providers will be able to formulate a more personalized treatment plan^75^. Several studies have successfully employed integrative multi-omics approaches to investigate novel mechanisms and plasma biomarkers associated with cardiovascular diseases, ultimately speeding up the identification of new therapeutic targets and pathways^76^. These studies serve as evidence that sophisticated integration techniques can yield dependable biological signals across various molecular levels and phenotypes^76^.

Our research underscores the critical need for an integrative approach that combines gene variant data with clinical information. We employed a multifaceted analysis, including functional mutation, splice variant, variant distribution, and divergence analysis, to discern the significance and prevalence of variants linked to well-studied genes associated with HF and CVD. Our variant analysis revealed the significance of additional genes, such as *ACE*, *CALD1*, *TEK,* and *TRPV1*. Among HF and other CVD genes, we observed that mutations in introns, the 5' flank, 3' UTR, and 3' flank regions were the most prevalent. Although missense mutations were infrequent, they were more likely to exert a functional impact. By employing JS-MA, we pinpointed *NPPC*, *ADRB2*, *ADBR1*, *MYH6*, *PLN*, *HBA1*, and *FADD* as the genes exhibiting the highest degree of variability. Previously, we have examined state-of-the art genomic approaches to identify and investigate genes associated with atrial fibrillation (AF) and HF susceptibility^23^. We found multiple genes such as *PLN*^77^, *MYH6*^77^, *NPPA*^77^*,* and *MYH7*^78^ to be significant, all of which were discovered to be notable in this study as well. The wide range of patients from various ages, ethnicities, demographics, and geographic locations as well as the variety of methods from these previous studies contributes to a randomized sample size^23^.

We expanded our research regarding these significant genes by exploring the clinical relevance of gene expression by leveraging RNA-seq data^30,79^. Our analysis focused on discerning the disparities between healthy and afflicted conditions, aiming to gain insights into the underlying disease pathology. We performed age and gender-based analyses to further understand shared and unique expressions across different ethnic and racial profiles^30,79^. Our previous and current studies have uncovered *ACE* to be a critical gene in CVD etiology and progression across all age groups. These findings hold significant importance for future research endeavors, as they indicate the opportunity to delve deeper into these genes opening a novel avenue that emphasizes a more personalized approach to therapy and treatment. The findings from previous studies corroborate our current results in this study. In conjunction, the variety of analyses performed including variant and prevalent analysis, functional mutation analysis, splice, and divergence analysis identified similar patterns and notable genes which suggests other confounding risk factors are not significant enough to overturn the conclusions reached in our study.

A multitude of genomic and statistical studies have similarly utilized phenotypic attributes such as gender, age, ethnicity, and diagnoses to determine gene causality in disease advancement^49–51^. While the age at which patients developed HF, severity of disease, alcoholic cardiomyopathy, different aetiologies of their HF, treatments received are important risk factors, recent approaches now focus on the heritability component that supports the clinical manifestation of the disease^50,51^. In this study, we utilized a cohort of only adult and aging CVD patients with HF phenotype. The data centered on age, gender, ethnicity, medical details, and demographics and added controls as the sample size was targeted and specific utilizing the restriction method designed to mitigate the effects of other confounding factors^80^. Our claims are supported by cutting-edge research, leading us to conclude that these confounding risk factors can be ruled out from the context of our study and have little relevance to our overall findings. In the future, we hope to expand our cohort of our healthy controls and patient cohorts to investigate and solidify the association between significant genes and the development of HF and CVDs.

For cardiovascular genomic medicine to become both predictive and preventive, it is crucial to accurately assess the risk of associated disease, properly report the variants, and implement clinical management to prevent or reduce the disease. Currently, multi-omics data are not available in formats that are useful for the AI/ML analysis. In the future, AI/ML-ready genomic data sets should be more widely available to integrate AI/ML algorithms in predictive analysis. ML can help identify a predictive response and model clinical data for association of genetic variants to treatment outcomes in HF and other CVDs^75^. We can process large volumes of clinical and variant data to identify biomarkers or gene sets associated with chronic diseases and improve diagnosis. With greater availability of AI/ML-ready datasets, the genomic data can be analyzed on a deeper level, with implications both in predictive analysis as well as deep phenotyping^81^. Additionally, growing evidence now suggests that there might be a direct link between infectious oral diseases and CVDs. The proposed mechanisms that explain the correlation between these two diseases consist of predisposing and precipitating aspects such as genetic and environmental factors, medications, and the individual’s microbiome^82^, Further studies have suggested that maladaptive inflammatory reactivity, which may be influenced by SNPs in pathway genes, could act as pleiotropic genes and effect the link between oral infections and CVDs^83,84^.

## Conclusion

Our study emphasizes the importance of an integrative approach with gene variant and clinical data and utilizes functional mutation, splice, variant distribution, and divergence analysis to identify the significance and prevalence of variants associated with commonly investigated HF and CVD genes. Our variant analysis uncovered additional genes to be significant including *ACE, CALD1, TEK*, and *TRPV1*. We discovered intron, 5ʹ Flank, 3ʹ UTR, and 3ʹ Flank mutations to be the most common among HF and other CVD genes. Missense mutations were rare but more likely to have functional impact. We implemented JS-MA and identified *NPPC*, *ADRB2*, *ADBR1*, *MYH6*, *PLN, HBA1*, and FADD genes to have the highest variance. The identification of the functional impact of these mutations will help us understand CVD progression and pathophysiology. Further studies are needed to determine if the genes with notable mutations can be used as potential biomarkers to improve early diagnosis and disease prediction.

### Supplementary Information

Supplementary Legends.

Supplementary Information 2.

Supplementary Information 3.

## Data Availability

Processed variant data of genes associated with HF and other CVDs are attached in the supplementary material. All the source code reproducing the experiments of this study are available at GitHub, following web links: JWES <https://github.com/drzeeshanahmed/JWES-Variant>, and JSD-Variant-Distribution-Analysis <https://github.com/drzeeshanahmed/JSD-Variant-Distribution-Analysis>.

## Abbreviations

AI: Artificial intelligence
AF: Atrial fibrillation
AVD: Atheromatous vascular disease
BWA: Burrows–Wheeler aligner
CNV: Copy number variants
CAD: Coronary artery disease
CHD: Coronary heart disease
CVD: Cardiovascular disease
EHR: Electronic health records
ETL: Extraction, transfer, loading
Gal-3: Galectin-3
GATK: Genome analysis toolkit
GWAS: Genome-wide association studies
HF: Heart failure
IRB: Institutional review board
JWES: Java based whole genome/exome sequence data processing pipeline
JSD: Jensen–Shannon divergence
JS-MA: Jensen–Shannon divergence-based method
MAV-clic: Management, analysis, and visualization of clinical data
ML: Machine learning
PolyPhen-2: Polymorphism phenotyping v2
Pfam domains: Functional protein domains
QC: Quality checking
SIFT: Scale-invariant feature transform
SV: Structural variants
STR: Short tandem repeats
SERCA2a: Ca^2+^ATPase
WGS: Whole-genome-sequencing
