# Supplementary material for: Functional mutation, splice, distribution, and divergence analysis of impactful genes associated with heart failure and other cardiovascular diseases
Source: Sci Rep. 2023 Oct 5;13:16769. doi: 10.1038/s41598-023-44127-1 (PMC10556087; doi:10.1038/s41598-023-44127-1)
Supplement: Supplementary file 1 — Supplementary Legends. [file 41598_2023_44127_MOESM1_ESM.pdf]

Supplementary information

Supplementary material, combined PDF file includes:

- Supplementary material# 1: List and Jensen-Shannon Divergence (JSD) scores of genes associated with Heart Failure and other cardiovascular diseases (CVD).
- Supplementary material# 2: Functional mutation analysis for genes associated with Heart Failure (HF).
- Supplementary material# 3: Functional mutation analysis for genes associated with other cardiovascular diseases (CVD).

In addition, separate Excel files are attached including supplementary datasets, demographics and personal information of subjects, quality check (QC) report, calculated pLI scores, and details of identified variants:

- Supplementary material# 4: Processed variant data of genes associated with Heart Failure.
- Supplementary material# 5: Processed variant data of genes associated with other cardiovascular diseases (CVD).
- Supplementary material# 6: Demographics, gender, and age of subjects.
- Supplementary material# 7: Quality check (QC) report of WGS samples.
- Supplementary material# 8: Calculated pLI score for heart failure (HF).
- Supplementary material# 9: Calculated pLI score for other cardiovascular diseases.
- Supplementary material# 10: Variants details of genes associated with heart failure (HF) and other cardiovascular diseases (CVDs).
