# Supplementary material for: Functional mutation, splice, distribution, and divergence analysis of impactful genes associated with heart failure and other cardiovascular diseases
Source: Sci Rep. 2023 Oct 5;13:16769. doi: 10.1038/s41598-023-44127-1 (PMC10556087; doi:10.1038/s41598-023-44127-1)
Supplement: Supplementary file 2 — Supplementary Information 2. [file 41598_2023_44127_MOESM2_ESM.pdf]

Supplementary Material 1:

List and Jensen-Shannon Divergence (JSD) scores of genes associated with Heart Failure and other cardiovascular diseases (CVD).

Authors

Ishani Mhatre[1, †], Habiba Abdelhalim[1, †], William Degroat[1, †], Shreya Ashok[1, †], Bruce T. Liang[4,5], and Zeeshan Ahmed[1,2,3, *]

Affiliations

1. Institute for Health, Health Care Policy and Aging Research, Rutgers University, 112 Paterson St, New Brunswick, NJ, USA.

2. Department of Medicine, Robert Wood Johnson Medical School, Rutgers Biomedical and Health Sciences, 125 Paterson St, New Brunswick, NJ, USA.

3. Department of Genetics and Genome Sciences, UConn Health, 400 Farmington Ave, Farmington, CT, USA.

4. Pat and Jim Calhoun Cardiology Center, UConn Health, 263 Farmington Ave, Farmington, CT, USA.

5. UConn School of Medicine, University of Connecticut, 263 Farmington Ave, Farmington, CT, USA.

[†]Equally contributing first authors.

[*]**Corresponding author**: Zeeshan Ahmed, Rutgers Institute for Health, Health Care Policy and Aging Research, Rutgers University, 112 Paterson Street, New Brunswick, 08901, NJ, USA. (zahmed@ifh.rutgers.edu).

Gene	Diagnosis
ACE	HF
ADM	HF
ADRB1	HF
ADRB2	HF
AGTR1	HF
AGT	HF
AMPD1	HF
ANKRD1	HF
AQP2	HF
CDKN2B-AS1	HF
CORIN	HF
CRP	HF
CST3	HF
EDN1	HF
EDNRA	HF
EPO	HF
HOTAIR	HF
HSPB7	HF
IL6	HF
KNG1	HF
LGALS3	HF
LSINCT5	HF
MME	HF
MMP2	HF
MYBPC3	HF
MYH6	HF
MYH7	HF
NOS3	HF
NPPA	HF
NPPB	HF
NPPC	HF
NPR1	HF
NR3C2	HF
PIK3C2A	HF
PLN	HF
REN	HF
RP11-451G4.2	HF
TNF	HF

TUSC7	HF
UTS2	HF
VCL	HF
ATP2A2	CVD
CALD1	CVD
CD34	CVD
CD40LG	CVD
DDX41	CVD
ENO2	CVD
FADD	CVD
FGF23	CVD
FGF2	CVD
FLNA	CVD
GJB6	CVD
GLMN	CVD
HBA1	CVD
KANTR	CVD
LEMD3	CVD
MB	CVD
PDPN	CVD
SLC2A1	CVD
SMUG1	CVD
TAC1	CVD
TEK	CVD
TRPV1	CVD
ZBTB8OS	CVD

Supplementary table 1: List genes associated with Heart Failure and other cardiovascular diseases (CVD).

Gene	Jensen-Shannon Divergence (JSD) Score
NPPC	0.489386143
ADRB2	0.474849657
ADRB1	0.473323041
MYH6	0.45344052
PLN	0.449422738
NR3C2	0.423398079
CRP	0.420858893
CORIN	0.414735714
NPPB	0.411180853
KNG1	0.393599572
ADM	0.383235525
TNF	0.367897627
AMPD1	0.361960693
MME	0.359443367
VCL	0.352096065
NPPA	0.345066418
MYBPC3	0.341091003
CST3	0.333346426
HSPB7	0.310561276
MMP2	0.309186855
LGALS3	0.308032334
EPO	0.29714969
REN	0.294153832
PIK3C2A	0.265788743
NOS3	0.263612986
IL6	0.24757385
EDNRA	0.241943703
AQP2	0.222224122
NPR1	0.21206172
UTS2	0.199935839
AGTR1	0.193560931
EDN1	0.191047996
ANKRD1	0.187290783
ACE	0.180822022
MYH7	0.162355887
AGT	0.094501279

Supplementary table 2: Jensen-Shannon Divergence (JSD) scores of genes associated with Heart Failure.

Gene	Jensen-Shannon Divergence (JSD) Score
HBA1	0.493785787
FADD	0.425903894
ENO2	0.328587929
GLMN	0.298883702
FLNA	0.293700839
CD40LG	0.280475233
FGF2	0.278549523
TAC1	0.268202974
CD34	0.254588171
DDX41	0.254338183
ZBTB8OS	0.250464325
SLC2A1	0.244988925
CALD1	0.236284079
TEK	0.213890777
PDPN	0.204408995
ATP2A2	0.167697361
LEMD3	0.167643627
MB	0.163000912
GJB6	0.155699384
FGF23	0.146866634
SMUG1	0.131022143
KANTR	0.107840684
TRPV1	0.092853216

Supplementary table 3: Jensen-Shannon Divergence (JSD) scores of genes associated with other cardiovascular diseases (CVD).

Supplementary Material 2:

Variant and functional mutation analysis for genes associated with Heart Failure (HF)

Authors

Ishani Mhatre[1, †], Habiba Abdelhalim[1, †], William Degroat[1, †], Shreya Ashok[1, †], Bruce T. Liang[4,5], and Zeeshan Ahmed[1,2,3, *]

Affiliations

1. Institute for Health, Health Care Policy and Aging Research, Rutgers University, 112 Paterson St, New Brunswick, NJ, USA.

2. Department of Medicine, Robert Wood Johnson Medical School, Rutgers Biomedical and Health Sciences, 125 Paterson St, New Brunswick, NJ, USA.

3. Department of Genetics and Genome Sciences, UConn Health, 400 Farmington Ave, Farmington, CT, USA.

4. Pat and Jim Calhoun Cardiology Center, UConn Health, 263 Farmington Ave, Farmington, CT, USA.

5. UConn School of Medicine, University of Connecticut, 263 Farmington Ave, Farmington, CT, USA.

[†]Equally contributing first authors.

[*]**Corresponding author**: Zeeshan Ahmed, Rutgers Institute for Health, Health Care Policy and Aging Research, Rutgers University, 112 Paterson Street, New Brunswick, 08901, NJ, USA. (zahmed@ifh.rutgers.edu).

Variant and functional mutation analysis for genes associated with Heart Failure (HF)

ACE: An enzyme that converts angiotensin I into angiotensin II and inactivates bradykinin, both of which ultimately cause an increase in blood pressure [1]. Certain mutations (silent and benign or harmful) in the ACE gene have been shown to increase the levels of ACE in individuals and lead to CVDs such as a myocardial infarction [2]. Specifically, a deletion allele also called the D allele has been associated with HF, high blood pressure, and non-CVDs such as complications with diabetes and asthma [3].

We plotted mutations on a lollipop graph based on specific positions of the amino acid sequence of ACE using MutationMapper [4]. A total of 247 mutations were mapped onto the sequence, 12 of which are missense mutations, 4 of which are splice mutations, 120 of which are intron mutations, 15 of which are silent mutations, 24 of which are 5' flank mutations, 66 of which are 3' flank mutations, and 6 of which are 3' UTR mutations. SIFT and PolyPhen-2 can be used to predict both loss-of-function and gain-of-function mutations as potential biomarkers [5]. Mutations with functional impact were given scores ranking their impact based on Scale-Invariant Feature Transform (SIFT) [6, 7, 8] and Polymorphism Phenotyping v2 (PolyPhen-2) [9]. Five missense mutations were found to have some negative impact on the function of the protein: they were mapped to W69G, N74T, I75S, T76P, and E767K. The mutations mapped to W69G and N74T had high functional impact (score of deleterious impact by SIFT, and a score of probably damaging by PolyPhen-2). The other three mutations all had medium functional impact, scored by mutation assessor (score of deleterious impact by SIFT, and a score of possibly damaging by PolyPhen-2). A total of 60 mutations failed to be mapped onto the lollipop graph.

ADM: A hormone produced by multiple tissues including the adrenal medulla and the heart. ADM plays a role in controlling blood pressure because it widens blood vessels which causes blood pressure to decline [10]. We plotted known mutations of ADM onto the amino acid sequence and found 51 mapped mutations [4]. ADM, there was only 1 missense mutation. 28 were 5' flank mutations, 3 were intron mutations, 16 were 3' flank mutations, 2 were 3' UTR mutations, and 1 was a silent mutation. SIFT and PolyPhen-2 scores were used to provide rankings for the negative impact of mutations [5]. The missense mutation had an overall low functional impact (score of 0.14, tolerated impact by SIFT [6, 7, 8] and a score of 0.83, possibly damaging by PolyPhen-2) [9]. For ADM, 3' and 5' flank mutations were the most numerous mutations, and these both 5' and 3' flank regions have been known to increase transcription

[10] and this may relate to the fact that ADM is found to be high in a few patients with HF and CVDs. A total of 29 mutations failed to be mapped onto the lollipop graph.

ADRB1: A receptor found mostly in the heart. Compounds such as norepinephrine and epinephrine can cause both heart rate and contractility to increase by binding to ADRB1 [11]. After plotting mutations of ADRB1 on a lollipop graph, we were able to map a total of 81 genes onto the amino acid sequence using MutationMapper [4]. There a total of 5 missense mutations, 5 silent mutations, 30 were 5' flank mutations, 31 were 3' flank mutations, 8 were 3' UTR mutations, and 2 were 5' UTR mutations. SIFT and PolyPhen-2 scores were used to provide rankings for the negative impact of mutations [5]. The missense mutation that was mapped to N369S had a high functional impact score (deleterious impact by SIFT and probably damaging impact by PolyPhen-2). Three other missense mutations, mapped to G389R, V5A, and S49G respectively, had low functional impact scores and were labeled as benign by PolyPhen-2. The missense mutations mapped on G389V had a deleterious impact by SIFT [6, 7, 8] and benign impact by PolyPhen-2 [9]. Overall, this gene most had 5' flank mutations and 3' flank mutations. A total of 1779 failed mutations were unable to be mapped. The number of mutations unable to be annotated was greater than the mutations mapped onto the graph.

ADRB2: A receptor with a similar function to ADRB1, it also causes both vascular smooth muscle and bronchial smooth muscle to widen [11]. By plotting mutations of ADRB1 on a lollipop graph, a total of 81 genes were mapped onto the amino acid sequence [4]. Of the 81 genes, 5 were missense mutations, 4 silent mutations, 38 were 5' flank mutations, 29 were 3' flank mutations, 2 were 3' UTR mutations, and 3 were 5' UTR mutations. SIFT and PolyPhen-2 scores were used to provide rankings for the negative impact of mutations [5]. The missense mutation that was mapped to S22OC had a low functional impact score (tolerated impact by SIFT and possibly damaging impact by PolyPhen-2) [6, 7, 8, 9]. Four of the missense mutations (mapped to G16R, E27Q, Q3H, and T164) had low functional impact scores and were labeled as benign by PolyPhen-2 [9]. ADRB2 most had 5' flank mutations and 3' flank mutations. A total of 1866 failed mutations were unable to be mapped. The number of mutations unable to be annotated was greater than the mutations mapped onto the graph.

AGTR1: A protein that acts as a target receptor for angiotensin II, causing an increase in blood pressure. A higher level of activity of AGTR1 has been linked to CVDs and high blood pressure [12, 13]. We plotted mutations on a lollipop graph based on specific positions of the amino acid sequence of AGTR1 [4]. 343 mutations were mapped: 257 were intron mutations, 2 silent mutations, 40 were 5' flank mutations, 31 were 3' flank mutations, 12 were 3' UTR mutations, and 1 was 5' UTR mutation. No information regarding the functional impact score for any of

these mutations was provided. The most common mutation type was intron mutation. A total of 479 failed mutations were unable to be mapped. The number of mutations unable to be annotated was greater than the mutations mapped onto the graph.

AGT: A protein used to produce angiotensin II through various enzymes, AGT plays a major role in the renin-angiotensin system and angiotensin II is known to increase blood pressure [14, 15, 16]. We mapped mutations on a lollipop graph based on specific positions of the amino acid sequence of AGT [4]. A total of 135 mutations were mapped. Of those mutations, 3 were missense mutations, 67 were intron mutations, 2 silent mutations, 37 were 5' flank mutations, 24 were 3' flank mutations, 2 were 3' UTR mutations. Scores ranking negative impact were provided based on SIFT [6, 7, 8] and PolyPhen-2 [9]. One missense mutation, mapped to T207M had a high functional impact (score 0.01, deleterious impact by SIFT and score 1.00, probably damaging impact by PolyPhen-2). The missense mutation mapped to A4P had medium to low functional impact (score 0.02, deleterious impact by SIFT and score 0.14, benign by PolyPhen-2). The last missense mutation, mapped to M259T had low functional impact (tolerated by SIFT and benign by PolyPhen-2). Overall, the most common type of mutation was intron mutation. A total of 718 failed mutations were unable to be mapped. The number of mutations unable to be annotated was greater than the mutations mapped onto the graph.

AMPD1: A gene associated with the normal function of skeletal muscle; a specific mutation in AMPD1 has been linked to musculoskeletal problems [17]. We plotted mutations of AMPD1 on a lollipop graph onto the amino acid sequence using MutationMapper to identify the functional impact for each mutation [4, 5]. 119 mutations were mapped and from those 4 were missense mutations, 1 was a truncating (nonsense) mutation, 1 was a splice mutation, 103 were intron mutations, 1 was a silent mutation, and 9 were 5' flank mutations. Scores ranking negative impact were provided based on SIFT [6, 7, 8] and PolyPhen-2 [9]. The two missense mutations mapped to P81L and K3201 had a deleterious impact by SIFT and probably damaging impact by PolyPhen-2. The missense mutation mapped to M3101 had medium functional impact (deleterious impact by SIFT and possibly damaging impact by PolyPhen-2). The last missense mutation, mapped to M662I, had low functional impact, and was labeled as benign. A total of 78 mutations failed to be mapped onto the lollipop graph.

ANKRD1: Codes for a protein called cardiac ankyrin repeating protein (CARP) in which increased levels of this protein is associated with HF [18]. We plotted mutations on a lollipop graph based on specific positions of the amino acid sequence of ANKRD1 [4]. A total of 81 mutations were mapped: 3 were missense mutations, 29 were 3' flank mutations, 15 were 5'

flank mutations, 1 was a 5' UTR mutation, 2 were 3' UTR mutations, and 31 were intron mutations. Scores ranking negative impact were provided based on SIFT [6, 7, 8] and PolyPhen-2 [9]. The missense mutations mapped to A276V had a low functional impact (score 0.02, deleterious impact by SIFT and score 0.27, benign impact by PolyPhen-2). No additional information regarding functional impact for the other missense mutations was provided. The most common types of mutations were intron and 3' flank mutations. A total of 61 mutations failed to be mapped onto the lollipop graph.

AQP2: A protein channel that assists in storing water in the body through the kidneys and is heavily influenced by anti-diuretic hormone [19, 20]. Using the cBioPortal Mutation Mapper [4], we plotted a total of 82 mutations on a lollipop graph based on specific positions of the amino acid sequence of ANKRD1. Of those 82 mutations, 1 was a splice mutation, 8 were 3' flank mutations, 25 were 5' flank mutations, 1 was a 5' UTR mutation, 17 were 3' UTR mutations, 28 were intron mutations, and 2 were silent mutations. No information was provided regarding functional impact for these mutations was provided. Overall, the most common types of mutations were intron and 5' flank mutations. A total of 85 failed mutations were unable to be mapped. The number of mutations unable to be annotated was greater than the mutations mapped onto the graph.

CORIN: A protein which generates the atrial natriuretic peptide (ANP) hormone which acts to lower blood pressure. Lower levels of ANP are associated with high blood pressure [21, 22]. We plotted mutations on a lollipop graph based on specific positions of the amino acid sequence of CORIN [4]. A total of 1275 mutations were mapped onto the sequence: 5 of which are missense mutations, 2 of which are splice mutations, 1,234 of which are intron mutations, 6 of which are silent mutations, 17 of which are 5' flank mutations, 2 of which are 3' flank mutations, and 9 of which are 3' UTR mutations. Scores ranking negative impact were provided based on SIFT [6, 7, 8] and PolyPhen-2 [9]. 3 missense mutations mapped to H525R, D574E, C13Y had low functional impact score (ranging from tolerated to tolerated-low-confidence by SIFT and benign impact by PolyPhen-2). One mutation, mapped to S683C, had deleterious impact by SIFT and possibly damaging by PolyPhen-2. The last missense mutation, mapped to R45W, had a higher functional impact (delirious low confidence impact by SIFT and probably damaging by PolyPhen-2). A total of 404 mutations failed to be mapped onto the lollipop graph.

CRP: A protein composed of liver cells and increased levels of CRP have been linked to CVDs [23]. We plotted mutations on a lollipop graph based on specific positions of the amino acid sequence of CRP [4]. A total of 50 mutations were mapped onto the sequence. Of those mutations, only 1 was a missense mutation, 2 were intron mutations, 2 were silent mutations,

32 were 5’ flank mutations, 8 were 3’ flank mutations, and 5 of which are 3’ UTR mutations. The missense mutation was mapped to F164L and had medium functional impact score (deleterious by SIFT [6, 7, 8] and possibly damaging by PolyPhen-2) [9]. The most common mutation type was the 5’ flank mutation. A total of 291 mutations were unable to be mapped. The number of mutations unable to be annotated was greater than the mutations mapped onto the graph.

CST3: It is a protein known as cystatin C and the main function is to control different types of chemical reactions by blocking enzymes. Based on past epidemiological studies, high circulating cystatin C is associated with risk of CVDs including heart failure and stroke [24, 25, 26]. Using the cBioPortal Mutation Mapper [4], we plotted mutations on a lollipop graph based on specific positions of the amino acid sequence of CST3. A total of 128 mutations were mapped. 2 of these mutations were missense, 16 were intron mutations, 2 were silent mutations, 33 were 5’ flank mutations, 67 were 3’ flank mutations, 2 were 5’ UTR mutations, and 6 were 3’ UTR mutations. The two missense mutations are mapped to A25T and G30S. Both had low functional impact (tolerated impact by SIFT and benign by PolyPhen-2) [5]. A total of 254 mutations were unable to be mapped. The number of mutations unable to be annotated was greater than the mutations mapped onto the graph.

EDN1: A protein that causes blood vessels to widen or narrow depending on the receptor it binds to [27]. We plotted known mutations of EDN1 onto the amino acid sequence and mapped 108 mutations [4]. There was only 1 missense mutation. 32 were 5’ flank mutations, 33 were intron mutations, 38 were 3’ flank mutations, 3 were 3’ UTR mutations, and 1 was a silent mutation. SIFT and PolyPhen-2 scores were used to rank the functional impact [5]. The missense mutation was mapped to K198N and had a low functional impact score (score 0.06, tolerated by SIFT and score 0.07, benign by PolyPhen-2). The three most common types were 3’ flank mutations, intron mutations, and 5’ flank mutations. A total of 1320 mutations were unable to be mapped. The number of mutations unable to be annotated was greater than the mutations mapped onto the graph.

EDNRA: A protein receptor found mostly in the vascular system, which causes blood vessels to narrow anytime its agonist, endothelin 1, binds to it [28]. Using cBioPortal Mutation Mapper to create lollipop graphs, we mapped 364 mutations of EDNRA onto the amino acid sequence [4]. Of the 364 mutations, 296 were intron mutations, 34 were 5’ flank mutations, 21 were 3’ flank mutations, 3 were 5’ UTR mutations, 8 were 3’ UTR mutations, and 2 were silent mutations. There were no missense mutations mapped. Furthermore, no information was provided for the functional impact of the mapped mutations. A total of 438 mutations were

unable to be mapped. The number of mutations unable to be annotated was greater than the mutations mapped onto the graph.

EPO: A hormone that helps produce red blood cells in the body. Abnormal amounts of EPO in patients have been associated with HF through prior studies [29, 30]. We plotted mutations on a lollipop graph based on specific positions of the amino acid sequence of EPO [4]. A total of 56 mutations were mapped onto the sequence, 9 of which are intron mutations, 25 of which are 5' flank mutations, 15 of which are 3' flank mutations, 3 of which are 5' UTR mutations, and 4 of which are 3' UTR mutations. There were no missense mutations mapped. Furthermore, no information was provided for the functional impact of the mapped mutations. A total of 124 mutations were unable to be mapped. The number of mutations unable to be annotated was greater than the mutations mapped onto the graph.

HSPB7: A protein found in the heart which contributes to actin formation. Mutations found in the HSPB7 gene have been linked to HF [31]. Using cBioPortal Mutation Mapper to create lollipop graphs, we mapped 158 mutations of HSPB7 onto the amino acid sequence [4]. Of the 158 mutations, 1 was a missense mutation, 19 were intron mutations, 66 were 5' flank mutations, 46 were 3' flank mutations, 3 were 5' UTR mutations, 19 were 3' UTR mutations, and 4 were silent mutations. SIFT and PolyPhen-2 scores were used to rank the functional impact [5]. The missense mutations are mapped to Q162P and have a low functional impact (score 0.27, tolerated by SIFT and score 0.19, benign by PolyPhen-2). No other information was provided regarding functional impact of the mutations. Overall, the most common mutation types were 5' flank and 3' flank mutations. A total of 14 mutations failed to be mapped onto the lollipop graph.

IL6: A cytokine protein that leads to increased inflammation. High levels of IL6 have been linked to HF [32, 33]. We plotted mutations on a lollipop graph based on specific positions of the amino acid sequence of IL6 [4]. A total of 71 mutations were mapped onto the sequence: 2 of which are missense mutations, 22 of which are intron mutations, 1 of which is a silent mutation, 15 of which are 5' flank mutations, and 31 of which are 3' flank mutations. The two missense mutations are mapped to P32S and D162E. SIFT and PolyPhen-2 scores were used to rank the functional impact [5]. Both mutations have a low functional impact (tolerated impact by SIFT and benign by PolyPhen-2). A total of 95 mutations failed to be mapped onto the lollipop graph.

KNG1: Codes for proteins that can affect blood pressure. Mutations found in KNG1 have been linked to high blood pressure and HF [34]. We plotted mutations on a lollipop graph based on specific positions of the amino acid sequence of KNG1 [4]. A total of 29 mutations were

mapped onto the sequence. Of those mutations, 3 were missense mutations, 1 was a truncating (nonsense) mutation, 1 was a splice mutation, 15 were intron mutations, 4 were silent mutations, 2 were 5' UTR mutations, 2 were 3' flank mutations, and 1 was a 3' UTR mutation. SIFT and PolyPhen-2 scores were used to rank the functional impact [5]. The missense mutation mapped to M178T had the lowest functional impact (tolerated impact by SIFT and benign by PolyPhen-2). The second missense mutation was mapped to I197M. This mutation had a higher functional impact (deleterious impact by SIFT and possibly damaging by PolyPhen-2). The third missense, mapped to T400K, also had a low functional impact (tolerated impact by SIFT and possibly damaging by PolyPhen-2). A total of 307 mutations were unable to be mapped. The number of mutations unable to be annotated was greater than the mutations mapped onto the graph.

LGALS3: It is a gene that codes for Galectin-3 (Gal-3). This protein plays an important role in cell proliferation, adhesion, differentiation, and apoptosis. Recent studies have linked Gal-3 levels to organ systems and organ health with an increase in Gal-3 being linked to fibrotic and inflammatory diseases [35]. We plotted mutations on a lollipop graph based on specific positions of the amino acid sequence of LGALS3 [4]. A total of 150 mutations were mapped onto the sequence. Of those mutations, 4 were missense mutations, 1 was a splice mutation, 83 were intron mutations, 61 were 5' flank mutations, 1 was 5' UTR mutations. SIFT and PolyPhen-2 scores were used to rank the functional impact [5]. The missense mutation mapped to P64H had a high functional impact (deleterious by SIFT and probably damaging by PolyPhen-2). The other two missense mutations were mapped to T98P and R183K. Both had low functional impact (tolerated impact by SIFT and benign by PolyPhen-2). No information regarding the function impact of the last missense mutation (mapped at Q201H) was given. There was also no additional information for the functional impact of the other mutations either. A total of 474 mutations were unable to be mapped. The number of mutations unable to be annotated was greater than the mutations mapped onto the graph.

LSINCT5: No graph produced.

MME: A protein that is a known marker for diagnosis of human acute lymphocytic leukemia, neprilysin is a membrane-bound enzyme. Trials have demonstrated HF patients treated with an angiotensin receptor neprilysin inhibitor lived longer without the need to be hospitalized [36]. We plotted mutations on a lollipop graph based on specific positions of the amino acid sequence of MME [4]. A total of 1044 mutations were mapped onto the sequence. Of those mutations, 3 were missense mutations, 3 were splice mutations, 953 were intron mutations, 63 were 5' flank mutations, 1 was a 5' UTR mutation, 1 was a 3' flank mutation, 18 were 3'

UTR mutations, and 2 were silent mutations. The missense mutation mapped to M8V had a lower functional impact (deleterious by SIFT and benign by PolyPhen-2) [5]. The second missense mutation mapped to Y347C had a medium functional impact (deleterious by SIFT and possibly damaging by PolyPhen-2). The third missense mutation mapped to I149V had high functional impact (deleterious by SIFT and probably damaging by PolyPhen-2). A total of 1096 mutations were unable to be mapped. The number of mutations unable to be annotated was greater than the mutations mapped onto the graph.

MMP2: Generates a protein that helps break down components of the extracellular matrix. High levels of MMP2 have been linked to HF [37]. We plotted mutations on a lollipop graph based on specific positions of the amino acid sequence of MME [4]. A total of 546 mutations were mapped onto the sequence. Of those mutations, 1 was a missense mutation, 1 was a splice mutation, 456 were intron mutations, 68 were 5' flank mutations, 2 were 5' UTR mutations, 9 were 3' UTR mutations, and 9 were silent mutations. The missense mutation was mapped to E166K, and it had a lower functional impact (tolerated by SIFT [6, 7, 8] and benign by PolyPhen-2) [9]. No other functional impact scores were provided for the other mutations. A total of 741 mutations were unable to be mapped. The number of mutations unable to be annotated was greater than the mutations mapped onto the graph.

MYBPC3: A protein found in the heart muscle to help contract. Mutations in this gene have been proven to be linked to HF [38]. We plotted mutations on a lollipop graph based on specific positions of the amino acid sequence of MYBPC3 [4]. A total of 104 mutations were mapped onto the sequence. Of those mutations, 9 were missense mutations, 1 was a truncating mutation, 1 was a splice mutation, 72 were intron mutations, 11 were 5' flank mutations, and 10 were silent mutations. SIFT and PolyPhen-2 scores were used to rank the functional impact [5]. The missense mutation mapped to M8V had a lower functional impact (deleterious by SIFT and benign by PolyPhen-2). The missense mutations were mapped to V896M, S236G, A833V, V158M, E619K, A562V, R326Q, E542Q, and P794L. Most of these mutations had low to medium functional scores, ranging from tolerated to deleterious by SIFT and benign to probably damaging by PolyPhen-2. One mutation, mapped to R326Q, had a high functional impact score which was deleterious by SIFT and probably damaging by PolyPhen-2. A total of 84 failed to be mapped onto the lollipop graph.

MYH6: Acts as a component of the myosin protein found in the heart. Mutations in MYH6 have been associated with cardiovascular dysfunction and HF [39]. We plotted mutations on a lollipop graph based on specific positions of the amino acid sequence of MYH6 [4]. A total of 89 mutations were mapped onto the sequence. Of those mutations, 3 were missense

mutations, 1 was a splice mutation, 74 were intron mutations, and 11 were silent mutations. SIFT and PolyPhen-2 scores were used to rank the functional impact [5]. The missense mutation mapped to D377E had a low to medium functional impact (deleterious by SIFT and benign by PolyPhen-2). The second missense mutation mapped to G56R had a high functional impact (deleterious by SIFT and probably damaging by PolyPhen-2). The third missense mutation mapped to D208N had the lowest functional impact (tolerated by SIFT and probably damaging by PolyPhen-2). A total of 51 failed to be mapped onto the lollipop graph.

MYH7: Similar to MYH6, also codes for a protein which acts as a component of the myosin protein in the heart. Mutations in MYH7 have been linked to an enlarged heart and other CVDs [40, 41]. We plotted mutations on a lollipop graph based on specific positions of the amino acid sequence of MYH7 [4]. A total of 95 mutations were mapped onto the sequence. Of those mutations, 4 were missense mutations, 5 were splice mutations, 105 were intron mutations, 35 were 5' flank mutations, 29 was a 3' flank mutation, 1 was a 3' UTR mutations, and 16 were silent mutations. The missense mutations were mapped to S1491C, N1327K, C1748Y, and E949V. Most mutations had a low to medium functional impact. The mutation mapped to E949V had high functional impact (deleterious by SIFT and probably damaging by PolyPhen-2). A total of 231 mutations were unable to be mapped. The number of mutations unable to be annotated was greater than the mutations mapped onto the graph.

NOS3: Generates a protein largely found in blood vessels which helps produce nitric oxide. Lower levels of NOS3 have been linked to HF [42]. We plotted mutations on a lollipop graph based on specific positions of the amino acid sequence of NOS3 [4]. A total of 199 mutations were mapped onto the sequence. Of those mutations, 7 were missense mutations, 143 were intron mutations, 39 were 5' flank mutations, 1 was a 5' UTR mutation, 1 was a 3' flank mutation, 2 were 3' UTR mutations, and 6 were silent mutations. The missense mutations were mapped to D298E, V827M, R764C, R112Q, R665H, R885M, and Q982L. Almost all mutations had a low functional impact score (tolerated by SIFT and benign by PolyPhen-2). One mutation, mapped to V827M, had a medium functional impact (score 0.05, deleterious by SIFT and score 0.67, possibly damaging by PolyPhen-2). A total of 116 mutations failed to be mapped onto the lollipop graph.

NPPA: Produces the atrial natriuretic peptide which acts to decrease blood pressure [43]. We plotted mutations on a lollipop graph based on specific positions of the amino acid sequence of NPPA [4]. A total of 49 mutations were mapped onto the sequence. Of those mutations, 3 were missense mutations, 1 was a truncating mutation, 16 were intron mutations, 25 were 5' flank mutations, , 4 were 3' UTR mutations. No information regarding the functional impact

score for any of these mutations was provided. The most common mutation type was 5' flank mutation. A total of 52 mutations were unable to be mapped. The number of mutations unable to be annotated was greater than the mutations mapped onto the graph.

NPPB: Generates a hormone found in the heart that helps to lower blood pressure. High levels of NPPB are associated wiyh HF [44]. We plotted mutations on a lollipop graph based on specific positions of the amino acid sequence of NPPB [4]. A total of 76 mutations were mapped onto the sequence. Of those mutations, 2 were missense mutations, 5 were intron mutations, 28 were 5' flank mutations, 40 were 3' flank mutations, and 1 was a silent mutation. The missense mutation mapped to R25L had low functional impact (tolerated by SIFT and benign by PolyPhen-2). The second missense mutation mapped to R133W had a slightly higher, but still low, functional impact (deleterious by SIzT and benign by PolyPhen-2). A total of 244 mutations were unable to be mapped. The number of mutations unable to be annotated was greater than the mutations mapped onto the graph.

NPPC: Generates a protein that may cause widening blood vessels, natriuresis, and a drop in blood pressure. HF patients have been found to have high levels of NPPC [45]. We plotted mutations on a lollipop graph based on specific positions of the amino acid sequence of NPPC [4]. A total of 60 mutations were mapped onto the sequence. Of those mutations, 1 was an intron mutation, 23 were 5' flank mutations, and 36 were 3' flank mutations. No information regarding the functional impact score for any of these mutations was provided. The most common mutation type was the 5' flank mutation. A total of 457 mutations were unable to be mapped. The number of mutations unable to be annotated was greater than the mutations mapped onto the graph.

NPR1: A vital receptor protein found in the heart along with many other organs. Mutations discovered in NPR1 have been associated with high blood pressure, HF, and other CVDs [46]. We plotted mutations on a lollipop graph based on specific positions of the amino acid sequence of NPR1 [4]. A total of 112 mutations were mapped onto the sequence and 5 were missense mutations, 53 were intron mutations, 13 were 5' flank mutations, 3 were 5' UTR mutations, 37 were 3' flank mutations, and 1 was a silent mutation. SIFT and PolyPhen-2 scores were used to rank the functional impact [5]. The missense mutations mapped to R439C and R6S had low functional impact (tolerated by SIFT and benign by PolyPhen-2). The third missense mutation mapped to P2L had a slightly higher functional impact (deleterious low confidence by SIFT and benign by PolyPhen-2. No additional information regarding the functional impact score for any of these mutations was provided. A total of 151 mutations

were unable to be mapped. The number of mutations unable to be annotated was greater than the mutations mapped onto the graph.

NR3C2: A protein known as mineralocorticoid which regulates the amount of water and salt levels in the body. Mineralocorticoid receptor antagonists (MRAs) prevent negative effects of aldosterone, a hormone known to cause deleterious effects on the cardiovascular system including vascular stiffening and stimulation of cardiac arrhythmias. The binding of aldosterone to NR3C2 has been linked to HF and other CVDs [47]. We plotted mutations on a lollipop graph based on specific positions of the amino acid sequence of *NR3C2*. A total of 2103 mutations were mapped onto the sequence. Of those mutations, 3 were missense mutations, 2 were splice mutations, 2,057 were intron mutations, 27 were 5' flank mutations, 1 was a 5' UTR mutation, 2 were 3' flank mutations, 6 were 3' UTR mutations, and 5 were silent mutations. The missense mutations were mapped to V180I, N444T, and S419L. The first mutation at V180I had a low functional impact score and was labeled as benign. The other two had a deleterious by SIFT and benign by PolyPhen-2. A total of 1079 mutations failed to be mapped onto the lollipop graph.

PIK3C2A: A protein that is part of the phosphoinositide 3-kinase (PI3K) family which deals with cell survival, proliferation, and migration. Mutations in *PIK3C2A* can lead to visual, skeletal, neurological, growth impairments as well as HF and other CVDs. A study revealed lower levels of *PIK3C2A* gene expression was found in patients with acute myocardial infarction [48, 49]. We plotted mutations on a lollipop graph based on specific positions of the amino acid sequence of PIK3C2A [4]. A total of 104 mutations were able to be mapped onto the sequence. From those mutations, 1 was a missense mutation, 1 was a splice mutation, 423 were intron mutations, 37 were 5' flank mutations, 2 were 5' UTR mutations, 33 were 3' flank mutations, 4 were 3' UTR mutations, 5 were silent mutations, and 1 was an RNA mutation. The missense mutation mapped to T1415A had a low functional impact (tolerated by SIFT and benign by PolyPhen-2). No additional information regarding the functional impact score for any of these mutations was provided. A total of 326 mutations were unable to be mapped. The number of mutations unable to be annotated was greater than the mutations mapped onto the graph.

PLN: A protein that affects contraction of heart muscle. Higher levels of activity have been associated with HF and other CVDs [50]. We plotted mutations on a lollipop graph based on specific positions of the amino acid sequence of PLN using MutationMapper [4]. A total of 58 mutations were mapped onto the sequence. Of those mutations, 49 were intron mutations and 9 were 3' UTR mutations. No information regarding the functional impact score for any of these mutations was provided. There were only two types of mutations and of those two,

intron mutations were the most common. A total of 46 mutations failed to be mapped onto the lollipop graph.

REN: Produces a hormone that helps regulate blood pressure and higher levels have been linked to HF and other CVDs [51, 52]. We plotted mutations on a lollipop graph based on specific positions of the amino acid sequence of REN using MutationMapper [4]. A total of 149 mutations were mapped onto the sequence. Of those mutations, 1 was a missense mutation, 1 was a splice mutation, 108 were intron mutations, 34 were 5' flank mutations, 3 were 5' UTR mutations, and 2 were silent mutations. The missense mutation was mapped to G217R and had a low functional impact (tolerated by SIFT and benign by PolyPhen-2). No further information regarding functional impact for the mutations was provided. A total of 150 mutations failed to be mapped onto the lollipop graph.

RP11-451G4.2: No graph produced.

TNF: A multifunctional cytokine protein that belongs to the TNF family. It is associated with cell proliferation, differentiation, and apoptosis. Prior studies have linked levels of proinflammatory cytokine, tumor necrosis factor-α (TNFα) with the severity of heart failure indicating it could be a potential biomarker [53]. We plotted mutations on a lollipop graph based on specific positions of the amino acid sequence of TNF [4]. A total of 24 mutations were mapped onto the sequence. Of those mutations, 7 were intron mutations, 15 were 5' flank mutations, and 2 were 3' UTR mutations. There were no missense mutations found. No additional information regarding functional impact for the mutations was provided. A total of 780 mutations failed to be mapped onto the lollipop graph.

TUSC7: No graph produced.

UTS2: Codes for a protein that causes blood vessels to narrow and high levels of it are linked to HF and other CVDs [54]. We plotted mutations on a lollipop graph based on specific positions of the amino acid sequence of UTS2 [4]. A total of 90 mutations were mapped onto the sequence. Of those mutations, 3 were missense mutations, 30 were intron mutations, 37 were 5' flank mutations, 19 were 3' flank mutations, and 1 was a silent mutation. The missense mutation mapped to I12T had no functional impact. Two 5' flank mutations had low functional impact and were labeled as benign. The missense mutation mapped to S74N had low functional impact (tolerated by SIFT and benign by PolyPhen-2). Another missense mutation mapped to S30F had a lightly higher, but still low, functional impact (deleterious by SIFT and benign by PolyPhen-2). A total of 806 mutations were unable to be mapped. The number of mutations unable to be annotated was greater than the mutations mapped onto the graph.

VCL: A protein coding gene that produces Vinculin, has a critical role in cell-cell and cell-matrix junctions in terms of rapidly developing replacement fibrosis and is a fundamental structural protein in cardiac myocytes. Previous studies found mutations in *VCL* in patients with cardiomyopathy and mutations that affect plasma level have been linked to HF [55]. We plotted mutations on a lollipop graph based on specific positions of the amino acid sequence of VCL using MutationMapper [4]. A total of 579 mutations were mapped onto the sequence. Of those mutations, 2 were missense mutations, 522 were intron mutations, 42 were 5' flank mutations, 1 was a 5' UTR mutation, 5 were 3' UTR mutations, and 7 were silent mutations. The missense mutation was mapped to R823Q and P943A. Both had low functional impact (tolerated by SIFT and benign by PolyPhen-2). No further information regarding the functional impact score for any of these mutations was provided. A total of 875 mutations were unable to be mapped. The number of mutations unable to be annotated was greater than the mutations mapped onto the graph.

References

1. Wong M. (2016). Angiotensin Converting Enzymes. Handbook of Hormones, 263–e29D-4. https://doi.org/10.1016/B978-0-12-801028-0.00254-3

2. Krege, J. H., Kim, H. S., Moyer, J. S., Jennette, J. C., Peng, L., Hiller, S. K., & Smithies, O. (1997). Angiotensin-converting enzyme gene mutations, blood pressures, and cardiovascular homeostasis. Hypertension (Dallas, Tex.: 1979), 29(1 Pt 2), 150–157. https://doi.org/10.1161/01.hyp.29.1.150

3. Gard P. R. (2010). Implications of the angiotensin converting enzyme gene insertion/deletion polymorphism in health and disease: a snapshot review. International journal of molecular epidemiology and genetics, 1(2), 145–157.

4. Vohra, S., & Biggin, P. C. (2013). Mutationmapper: a tool to aid the mapping of protein mutation data. *PloS one*, *8*(8), e71711. https://doi.org/10.1371/journal.pone.0071711

5. Flanagan, S. E., Patch, A. M., & Ellard, S. (2010). Using SIFT and PolyPhen to predict loss-of-function and gain-of-function mutations. *Genetic testing and molecular biomarkers*, *14*(4), 533–537. https://doi.org/10.1089/gtmb.2010.0036

6. Ng, P. C., & Henikoff, S. (2003). SIFT: Predicting amino acid changes that affect protein function. Nucleic acids research, 31(13), 3812–3814.

7. Sim, N. L., Kumar, P., Hu, J., Henikoff, S., Schneider, G., & Ng, P. C. (2012). SIFT web server: predicting effects of amino acid substitutions on proteins. Nucleic acids research, 40(Web Server issue), W452–W457.

8. Kumar, P., Henikoff, S., & Ng, P. C. (2009). Predicting the effects of coding non-synonymous variants on protein function using the SIFT algorithm. Nature protocols, 4(7), 1073–1081.

9. Adzhubei, I., Jordan, D. M., & Sunyaev, S. R. (2013). Predicting functional effect of human missense mutations using PolyPhen-2. Current protocols in human genetics, Chapter 7, Unit7.20.

10. Wong, H. K., Cheung, T. T., & Cheung, B. M. (2012). Adrenomedullin and cardiovascular diseases. JRSM cardiovascular disease, 1(5), cvd.2012.012003. https://doi.org/10.1258/cvd.2012.012003

11. Leineweber, K., & Heusch, G. (2009). Beta 1- and beta 2-adrenoceptor polymorphisms and cardiovascular diseases. British journal of pharmacology, 158(1), 61–69. https://doi.org/10.1111/j.1476-5381.2009.00187.x

12. Kawai, T., Forrester, S. J., O'Brien, S., Baggett, A., Rizzo, V., & Eguchi, S. (2017). AT1 receptor signaling pathways in the cardiovascular system. Pharmacological research, 125(Pt A), 4–13. https://doi.org/10.1016/j.phrs.2017.05.008

13. Singh, K. D., & Karnik, S. S. (2016). Angiotensin Receptors: Structure, Function, Signaling and Clinical Applications. Journal of cell signaling, 1(2), 111. https://doi.org/10.418/jcs.1000111

14. Lu, H., Cassis, L. A., Kooi, C. W., & Daugherty, A. (2016). Structure and functions of angiotensinogen. Hypertension research : official journal of the Japanese Society of Hypertension, 39(7), 492–500. https://doi.org/10.1038/hr.2016.17

15. Gribouval, O., Gonzales, M., Neuhaus, T., Aziza, J., Bieth, E., Laurent, N., Bouton, J. M., Feuillet, F., Makni, S., Ben Amar, H., Laube, G., Delezoide, A. L., Bouvier, R., Dijoud, F., Ollagnon-Roman, E., Roume, J., Joubert, M., Antignac, C., & Gubler, M. C. (2005). Mutations in genes in the renin-angiotensin system are associated with autosomal recessive renal tubular dysgenesis. Nature genetics, 37(9), 964–968. https://doi.org/10.1038/ng1623

16. Jeunemaitre, X., Soubrier, F., Kotelevtsev, Y. V., Lifton, R. P., Williams, C. S., Charru, A., Hunt, S. C., Hopkins, P. N., Williams, R. R., & Lalouel, J. M. (1992). Molecular basis of human hypertension: role of angiotensinogen. Cell, 71(1), 169–180. https://doi.org/10.1016/0092-8674(92)90275-h

17. Smolenski, R. T., Rybakowska, I., Turyn, J., Romaszko, P., Zabielska, M., Taegtmeyer, A., Słomińska, E. M., Kaletha, K. K., & Barton, P. J. (2014). AMP deaminase 1 gene polymorphism and heart disease-a genetic association that highlights new treatment. Cardiovascular drugs and therapy, 28(2), 183–189. https://doi.org/10.1007/s10557-013-6506-5

18. Zhang, N., Xie, X. J., & Wang, J. A. (2016). Multifunctional protein: cardiac ankyrin repeat protein. Journal of Zhejiang University. Science. B, 17(5), 333–341. https://doi.org/10.1631/jzus.B1500247

19. Radin, M. J., Yu, M. J., Stoedkilde, L., Miller, R. L., Hoffert, J. D., Frokiaer, J., Pisitkun, T., & Knepper, M. A. (2012). Aquaporin-2 regulation in health and disease. Veterinary clinical pathology, 41(4), 455–470. https://doi.org/10.1111/j.1939-165x.2012.00488.x

20. Xu, D. L., Martin, P. Y., Ohara, M., St John, J., Pattison, T., Meng, X., Morris, K., Kim, J. K., & Schrier, R. W. (1997). Upregulation of aquaporin-2 water channel expression in chronic heart failure rat. The Journal of clinical investigation, 99(7), 1500–1505. https://doi.org/10.1172/JCI119312

21. Yan, W., Wu, F., Morser, J., & Wu, Q. (2000). Corin, a transmembrane cardiac serine protease, acts as a pro-atrial natriuretic peptide-converting enzyme. Proceedings of the National Academy of Sciences of the United States of America, 97(15), 8525–8529. https://doi.org/10.1073/pnas.150149097

22. Wang, W., Liao, X., Fukuda, K., Knappe, S., Wu, F., Dries, D. L., Qin, J., & Wu, Q. (2008). Corin variant associated with hypertension and cardiac hypertrophy exhibits impaired zymogen activation and natriuretic peptide processing activity. Circulation research, 103(5), 502–508. https://doi.org/10.1161/CIRCRESAHA.108.177352

23. Singh, S. K., Suresh, M. V., Voleti, B., & Agrawal, A. (2008). The connection between C-reactive protein and atherosclerosis. Annals of medicine, 40(2), 110–120. https://doi.org/10.1080/07853890701749225

24. van der Laan, S. W., Fall, T., Soumaré, A., Teumer, A., Sedaghat, S., Baumert, J., Zabaneh, D., van Setten, J., Isgum, I., Galesloot, T. E., Arpegård, J., Amouyel, P., Trompet, S., Waldenberger, M., Dörr, M., Magnusson, P. K., Giedraitis, V., Larsson, A., Morris, A. P., Felix, J. F., … Asselbergs, F. W. (2016). Cystatin C and Cardiovascular Disease: A Mendelian Randomization Study. Journal of the American College of Cardiology, 68(9), 934–945. https://doi.org/10.1016/j.jacc.2016.05.092

25. Shlipak, M. G., Sarnak, M. J., Katz, R., Fried, L. F., Seliger, S. L., Newman, A. B., Siscovick, D. S., & Stehman-Breen, C. (2005). Cystatin C and the risk of death and cardiovascular events among elderly persons. The New England journal of medicine, 352(20), 2049–2060. https://doi.org/10.1056/NEJMoa043161

26. Ni, L., Lü, J., Hou, L. B., Yan, J. T., Fan, Q., Hui, R., Cianflone, K., Wang, W., & Wang, D. W. (2007). Cystatin C, associated with hemorrhagic and ischemic stroke, is a strong predictor

of the risk of cardiovascular events and death in Chinese. Stroke, 38(12), 3287–3288. https://doi.org/10.1161/STROKEAHA.107.489625

27. Stauffer, B. L., Westby, C. M., & DeSouza, C. A. (2008). Endothelin-1, aging and hypertension. Current opinion in cardiology, 23(4), 350–355. https://doi.org/10.1097/HCO.0b013e328302f3c6

28. Giannessi, D., Del Ry, S., & Vitale, R. L. (2001). The role of endothelins and their receptors in heart failure. Pharmacological research, 43(2), 111–126. https://doi.org/10.1006/phrs.2000.0118

29. Bunn H. F. (2013). Erythropoietin. Cold Spring Harbor perspectives in medicine, 3(3), a011619. https://doi.org/10.1101/cshperspect.a011619

30. Garimella, P. S., Katz, R., Patel, K. V., Kritchevsky, S. B., Parikh, C. R., Ix, J. H., Fried, L. F., Newman, A. B., Shlipak, M. G., Harris, T. B., Sarnak, M. J., & Health ABC Study (2016). Association of Serum Erythropoietin With Cardiovascular Events, Kidney Function Decline, and Mortality: The Health Aging and Body Composition Study. Circulation. Heart failure, 9(1), e002124. https://doi.org/10.1161/CIRCHEARTFAILURE.115.002124

31. Wu, T., Mu, Y., Bogomolovas, J., Fang, X., Veevers, J., Nowak, R. B., Pappas, C. T., Gregorio, C. C., Evans, S. M., Fowler, V. M., & Chen, J. (2017). HSPB7 is indispensable for heart development by modulating actin filament assembly. Proceedings of the National Academy of Sciences of the United States of America, 114(45), 11956–11961. https://doi.org/10.1073/pnas.1713763114

32. Uciechowski, P., & Dempke, W. (2020). Interleukin-6: A Masterplayer in the Cytokine Network. Oncology, 98(3), 131–137. https://doi.org/10.1159/000505099

33. Kanda, T., & Takahashi, T. (2004). Interleukin-6 and cardiovascular diseases. Japanese heart journal, 45(2), 183–193. https://doi.org/10.1536/jhj.45.183

34. Zhao, W., Wang, Y., Wang, L., Lu, X., Yang, W., Huang, J., Chen, S., & Gu, D. (2009). Gender-specific association between the kininogen 1 gene variants and essential hypertension in Chinese Han population. Journal of hypertension, 27(3), 484–490. https://doi.org/10.1097/hjh.0b013e32831e19f9

35. Amin, H. Z., Amin, L. Z., & Wijaya, I. P. (2017). Galectin-3: a novel biomarker for the prognosis of heart failure. Clujul medical (1957), 90(2), 129–132. https://doi.org/10.15386/cjmed-751

36. Bayés-Genís, A., Barallat, J., Galán, A., de Antonio, M., Domingo, M., Zamora, E., Urrutia, A., & Lupón, J. (2015). Soluble neprilysin is predictive of cardiovascular death and heart failure hospitalization in heart failure patients. Journal of the American College of Cardiology, 65(7), 657–665. https://doi.org/10.1016/j.jacc.2014.11.048

37. Yamazaki, T., Lee, J. D., Shimizu, H., Uzui, H., & Ueda, T. (2004). Circulating matrix metalloproteinase-2 is elevated in patients with congestive heart failure. European journal of heart failure, 6(1), 41–45. https://doi.org/10.1016/j.ejheart.2003.05.002

38. Sadayappan, S., & de Tombe, P. P. (2014). Cardiac myosin binding protein-C as a central target of cardiac sarcomere signaling: a special mini review series. Pflugers Archiv : European journal of physiology, 466(2), 195–200. https://doi.org/10.1007/s00424-013-1396-8

39. Hao, E., Zhang, G., Mu, L., Ma, N., & Wang, T. (2020). Establishment of a human MYH6 compound heterozygous knockout hESC line to model cardiomyopathy and congenital heart defects by CRISPR/Cas9 system. Stem cell research, 50, 102128. Advance online publication. https://doi.org/10.1016/j.scr.2020.102128

40. Bánfai, Z., Hadzsiev, K., Pál, E., Komlósi, K., Melegh, M., Balikó, L., & Melegh, B. (2017). Novel phenotypic variant in the MYH7 spectrum due to a stop-loss mutation in the C-terminal region: a case report. BMC medical genetics, 18(1), 105. https://doi.org/10.1186/s12881-017-0463-y

41. Tanjore, R., Rangaraju, A., Vadapalli, S., Remersu, S., Narsimhan, C., & Nallari, P. (2010). Genetic variations of β-MYH7 in hypertrophic cardiomyopathy and dilated cardiomyopathy. Indian journal of human genetics, 16(2), 67–71. https://doi.org/10.4103/0971-6866.69348

42. Carnicer, R., Crabtree, M. J., Sivakumaran, V., Casadei, B., & Kass, D. A. (2013). Nitric oxide synthases in heart failure. Antioxidants & redox signaling, 18(9), 1078–1099. https://doi.org/10.1089/ars.2012.4824

43. Song, W., Wang, H., & Wu, Q. (2015). Atrial natriuretic peptide in cardiovascular biology and disease (NPPA). Gene, 569(1), 1–6. https://doi.org/10.1016/j.gene.2015.06.029

44. Krishnaswami A. (2008). The role of B-type and other natriuretic peptides in health and disease. The Permanente journal, 12(4), 32–43. https://doi.org/10.7812/tpp/08-019

45. Lumsden, N. G., Khambata, R. S., & Hobbs, A. J. (2010). C-type natriuretic peptide (CNP): cardiovascular roles and potential as a therapeutic target. Current pharmaceutical design, 16(37), 4080–4088. https://doi.org/10.2174/138161210794519237

46. Potter, L. R., Yoder, A. R., Flora, D. R., Antos, L. K., & Dickey, D. M. (2009). Natriuretic peptides: their structures, receptors, physiologic functions and therapeutic applications. Handbook of experimental pharmacology, (191), 341–366. https://doi.org/10.1007/978-3-540-68964-5_15

47. Vizzardi, E., Regazzoni, V., Caretta, G., Gavazzoni, M., Sciatti, E., Bonadei, I., Trichaki, E., Raddino, R., & Metra, M. (2014). Mineralocorticoid receptor antagonist in heart failure:

Past, present and future perspectives. International journal of cardiology. Heart & vessels, 3, 6–14. https://doi.org/10.1016/j.ijchv.2014.03.005

48. Tiosano, D., Baris, H. N., Chen, A., Hitzert, M. M., Schueler, M., Gulluni, F., Wiesener, A., Bergua, A., Mory, A., Copeland, B., Gleeson, J. G., Rump, P., van Meer, H., Sival, D. A., Haucke, V., Kriwinsky, J., Knaup, K. X., Reis, A., Hauer, N. N., Hirsch, E., … Buchner, D. A. (2019). Mutations in PIK3C2A cause syndromic short stature, skeletal abnormalities, and cataracts associated with ciliary dysfunction. PLoS genetics, 15(4), e1008088. https://doi.org/10.1371/journal.pgen.1008088

49. Tan, B., Liu, M., Yang, Y., Liu, L., & Meng, F. (2019). Low expression of PIK3C2A gene: A potential biomarker to predict the risk of acute myocardial infarction. Medicine, 98(14), e15061. https://doi.org/10.1097/MD.0000000000015061

50. Chu, G., & Kranias, E. G. (2006). Phospholamban as a therapeutic modality in heart failure. Novartis Foundation symposium, 274, 156–276.

51. Persson P. B. (2003). Renin: origin, secretion and synthesis. The Journal of physiology, 552(Pt 3), 667–671. https://doi.org/10.1113/jphysiol.2003.049890

52. Unger, T., & Li, J. (2004). The role of the renin-angiotensin-aldosterone system in heart failure. Journal of the renin-angiotensin-aldosterone system : JRAAS, 5 Suppl 1, S7–S10. https://doi.org/10.3317/jraas.2004.024

53. Schumacher, S. M., & Naga Prasad, S. V. (2018). Tumor Necrosis Factor-α in Heart Failure: an Updated Review. Current cardiology reports, 20(11), 117. https://doi.org/10.1007/s11886-018-1067-7

54. Russell F. D. (2008). Urotensin II in cardiovascular regulation. Vascular health and risk management, 4(4), 775–785. https://doi.org/10.2147/vhrm.s1983

55. Zemljic-Harpf, A., Manso, A. M., & Ross, R. S. (2009). Vinculin and talin: focus on the myocardium. Journal of investigative medicine : the official publication of the American Federation for Clinical Research, 57(8), 849–855. https://doi.org/10.2310/JIM.0b013e3181c5e074

Supplementary Material 3:

Variant and functional mutation analysis for genes associated with other cardiovascular diseases (CVD)

Authors

Ishani Mhatre[1, †], Habiba Abdelhalim[1, †], William Degroat[1, †], Shreya Ashok[1, †], Bruce T. Liang[4,5], and Zeeshan Ahmed[1,2,3, *]

Affiliations

1. Institute for Health, Health Care Policy and Aging Research, Rutgers University, 112 Paterson St, New Brunswick, NJ, USA.

2. Department of Medicine, Robert Wood Johnson Medical School, Rutgers Biomedical and Health Sciences, 125 Paterson St, New Brunswick, NJ, USA.

3. Department of Genetics and Genome Sciences, UConn Health, 400 Farmington Ave, Farmington, CT, USA.

4. Pat and Jim Calhoun Cardiology Center, UConn Health, 263 Farmington Ave, Farmington, CT, USA.

5. UConn School of Medicine, University of Connecticut, 263 Farmington Ave, Farmington, CT, USA.

[†]Equally contributing first authors.

[*]**Corresponding author**: Zeeshan Ahmed, Rutgers Institute for Health, Health Care Policy and Aging Research, Rutgers University, 112 Paterson Street, New Brunswick, 08901, NJ, USA. (zahmed@ifh.rutgers.edu).

Variant and functional mutation analysis for genes associated with other cardiovascular diseases (CVD)

ATP2A2: The ATP2A2 gene codes for a specific enzyme that belongs to the ATPase family of enzymes and helps regulate the level of calcium ions in a cell. Calcium ions are integral for the regular function and development of cell life. A previous study has suggested restoring the expression of Ca^{2+}ATPase (SERCA2a) in patients with heart failure can correct abnormalities and improve electrical remodeling [1].

To analyze the ATP2A2 gene, we generated a lollipop plot of the ATP2A2 sequence data on cBioPortal's MutationMapper software [2]. SIFT and PolyPhen-2 can be used to predict both loss-of-function and gain-of-function mutations as potential biomarkers [3]. Mutations with functional impact were given scores ranking their impact based on Scale-Invariant Feature Transform (SIFT) [4, 5, 6] and Polymorphism Phenotyping v2 (PolyPhen-2) [7]. We found a total of 330 mutations mapped onto the ATP2A2 sequence. Of the 330 mutations, there were 2 splices, 270 introns, 6 5'UTR, 11 5'Flank, 14 3'UTR, 24 3'Flank, and 3 silent. No functional impact scores were retrieved for any of these mutations. MutationMapper software failed to annotate 478 mutations.

CALD1: Generates a protein that interacts with myosin in smooth muscle and affects. Mutations found in CALD1 are linked with CVDs [8, 9]. To analyze the CALD1 gene, we generated a lollipop plot of the CALD1 sequence data on cBioPortal's MutationMapper software [2]. We found a total of 1210 mutations mapped onto the CALD1 sequence. Of the 1210 mutations, there were 8 missense, 1100 introns, 1 5'UTR, 66 5'Flank, 7 3'UTR, 26 3'Flank, and 2 silent. Mutations with functional impact were given scores ranking their impact based on Scale-Invariant Feature Transform (SIFT) [4, 5, 6] and Polymorphism Phenotyping v2 (PolyPhen-2) [7]. Within the 8 missense mutations, the breakdown was 1 tolerated low confidence and benign, 2 deleterious and benign, 2 deleterious low confidence and benign, 1 deleterious and possibly damaging, 1 tolerated and benign, and 1 deleterious and probably damaging. MutationMapper failed to annotate 492 mutations.

CD34: A gene that helps generate red blood cells and assists in various other functions. Low levels of CD34 have been associated with multiple CVDs [10]. To analyze the CD34 gene, we generated a lollipop plot of the CD34 sequence data on cBioPortal's MutationMapper software [2]. We found a total of 182 mutations mapped onto the CD34 sequence. Of the 182 mutations, there were 1 missense, 1 splice, 109 introns, 30 5'Flank, 39 3'UTR, 1 3'Flank, and 1 silent. No functional impact scores were retrieved for any of these mutations. MutationMapper failed to annotate 701 mutations.

CD40LG: Generates a protein that causes inflammation when it binds to the CD40 receptor, and higher levels are associated with many CVDs [11]. To analyze the CD40LG gene, we generated a lollipop plot of the CD40LG sequence data on cBioPortal's MutationMapper software [2]. We found a total of 61 mutations mapped onto the CD40LG sequence. Of the 61 mutations, there were 32 introns, 18 5'Flank, 9 3'UTR, and 2 silent. No functional impact scores were retrieved for any of these mutations. MutationMapper failed to annotate 56 mutations.

DDX41: Codes for a protein that is involed in numerous aspects of RNA metabolism, helps stimulate interferon release, and binds to nucleic [12]. Mutations in DDX41 have been linked to acute myeloid leukemia and other CVDs [13, 14]. To analyze the DDX41 gene, we generated a lollipop plot of the DDX41 sequence data on cBioPortal's MutationMapper software [2]. We found a total of 26 mutations mapped onto the DDX41 sequence. Of the 26 mutations, there were 2 splices, 17 introns, 4 5'Flank, 2 3'UTR, and 1 silent. No functional impact scores were retrieved for any of these mutations. MutationMapper failed to annotate 13 mutations.

ENO2: A protein involved in glycolysis and primarily found in neurons, increased amounts of ENO2 are associated with lower survival rates of CVDs, specifically for cardiac arrest [15, 16]. To analyze the ENO2 gene, we generated a lollipop plot of the ENO2 sequence data on cBioPortal's MutationMapper software [2]. We found a total of 63 mutations mapped onto the ENO2 sequence. Of the 63 mutations, there were 1 missense, 1 splice, 51 introns, 1 5'UTR, 8 3'UTR, and 1 silent. The 1 missense mutation was classified as deleterious low confidence and benign. MutationMapper failed to annotate 79 mutations.

FADD: A protein that interacts with cell receptors and mediates signals that assist in the process of apoptosis. Past studies have suggested FADD plays an important role in post-ischemic heart failure and that further studies are needed to study if FADD can be used in gene therapy for heart failure treatment. Restricting the activity of FADD has been linked to improved outcomes of CVDs [17]. To analyze the FADD gene, we generated a lollipop plot of the FADD sequence data on cBioPortal's MutationMapper software [2]. We found a total of 70 mutations mapped onto the FADD sequence. Of the 70 mutations, there were 9 introns, 2 5'UTR, 36 5'Flank, 3 3'UTR, and 20 3'Flank. No functional impact scores were retrieved for any of these mutations. MutationMapper failed to annotate 706 mutations.

FGF23: Codes for a protein that helps control phosphate and 1,25-dihydroxyvitamin D levels, and high amounts of FGF23 are associated with CVDs [18]. To analyze the FGF23 gene, we generated a lollipop plot of the FGF23 sequence data on cBioPortal's MutationMapper software [2] We found a total of 125 mutations mapped onto the FGF23 sequence. Of the 125 mutations, there were 2 missense, 68 introns,

33 5’Flank, 5 3’UTR, and 17 3’Flank. Scores for negative impact were given to the mutations based on SIFT and PolyPhen-2 [3]. Within the 2 missense mutations, the breakdown was 1 tolerated low confidence and benign and 1 tolerated and benign. MutationMapper failed to annotate 451 mutations.

FGF2: Generates a protein involved in cell development, growth, and development of new blood vessels, high levels are linked to CVDs including abnormal amounts of fluid in the pericardium [18]. To analyze the FGF2 gene, we generated a lollipop plot of the FGF2 sequence data on cBioPortal’s MutationMapper software [2]. We found a total of 434 mutations mapped onto the FGF23 sequence. Of the 434 mutations, there were 394 introns, 37 5’Flank, and 3 silent. No functional impact scores were retrieved for any of these mutations. MutationMapper failed to annotate 694 mutations.

FLNA: A protein which affects both cell structure and movement. Mutations in FLNA are associated with morphological abnormalities of the heart and to CVDs [19]. The *FLNA* gene supports production of a protein known as filamin A, which helps build the cytoskeleton of a cell, essential for cellular structure, flexibility, movement, and breakdown of proteins. Mutations in FLNA can be associated with cardiac, especially in the left ventricular outflow tract [9].

To analyze the FLNA gene, we generated a lollipop plot of the FLNA sequence data on cBioPortal’s MutationMapper software [2]. We found a total of 113 mutations mapped onto the FLNA sequence. Of the 113 mutations, there were 2 splice, 6 missense, 82 introns, 2 3’UTR, 10 3’Flank, and 11 silent. No functional impact scores were retrieved for any of these mutations. MutationMapper failed to annotate 73 mutations.

GJB6: A protein known as a gap junction protein, it facilitates the movement of molecules and mutations in GJB6 are linked to increased difficulty with CVDs [20, 21]. To analyze the GJB6 gene, we generated a lollipop plot of the GJB6 sequence data on cBioPortal’s MutationMapper software [2]. We found a total of 175 mutations mapped onto the GJB6 sequence. Of the 175 mutations, there was 1 missense, 88 introns, 3 5’UTR, 42 5’Flank, 4 3’UTR, 36 3’Flank, and 1 silent. Scores based on SIFT and PolyPhen-2 were used to rank the functional impact of mutations [3]. The 1 missense mutation was classified as tolerated and benign. MutationMapper failed to annotate 2133 mutations.

GLMN: Codes for a protein has an essential role in developmeing blood vessels. Mutations in GLMN are linked to issues with vessel development and to CVDs [22]. To analyze the GLMN gene, we generated a lollipop plot of the GLMN sequence data on cBioPortal’s MutationMapper software [2]. We found a total of 182 mutations mapped onto the GLMN sequence. Of the 182 mutations, there were 3 missense, 1 splice, 172 introns, 1 5’Flank, 1 3’UTR, and 4 3’Flank. Scores based on SIFT and PolyPhen-2 were used to

rank the functional impact of mutations [3]. Within the 3 missense mutations, the breakdown was 2 deleterious and possibly damaging and 1 tolerated and possibly damaging. MutationMapper failed to annotate 96 mutations.

HBA1: An alpha-globin protein which is an important component of hemoglobin, a larger protein that brings oxygen to various cells and tissues in the body [23]. A previous study discovered hemoglobin levels outside of a control range were independently associated with coronary artery disease (CAD) [24]. The risk for CAD increased when hemoglobin levels were either greater than or equal to 17.0 g/dL or less than 15 g/dL [24].

To analyze the HBA1 gene, we generated a lollipop plot of the HBA2 sequence data on cBioPortal's MutationMapper software [2]. We found a total of 30 mutations mapped onto the HBA2 sequence, and the HBA2 sequence is identical to that of HBA1. Of the 30 mutations, there were 2 introns, 12 5'Flank, 1 3'UTR, and 15 3'Flank. No functional impact scores were retrieved for any of these mutations. MutationMapper failed to annotate 28 mutations.

KANTR: An mRNA molecule that does not generate a protein, mutations found in KANTR are associated with uncontrolled movements and are linked to CVDs [25]. To analyze the KANTR gene, we generated a lollipop plot of the KANTR sequence data on cBioPortal's MutationMapper software [2]. We found a total of 197 mutations mapped onto the KANTR sequence. Of the 197 mutations, there were 145 introns, 4 5'UTR, 17 5'Flank, 7 3'UTR, 20 3'Flank, 1 silent, and 3 RNA. No functional impact scores were retrieved for any of these mutations. MutationMapper failed to annotate 165 mutations.

LEMD3: A protein located in the inner nuclear membrance that interacts with Smad 2 and Smad 3 and provides instructions on how to regulate two chemical pathways in the nuclear envelope of the cell: the TGF-β or transforming growth factor beta pathway and the BMP or bone morphogenic protein pathway [26, 27]. Both pathways help with the control of cell proliferation, differentiation, and apoptosis. Mutations in *LEMD3* have been linked to CVDs and other diseases including osteopoikilosis, Buschke–Ollendorff syndrome and melorheostosis [27].

To analyze the LEMD3 gene, we generated a lollipop plot of the LEMD3 sequence data on cBioPortal's MutationMapper software. We found a total of 322 mutations mapped onto the LEMD3 sequence [2]. Of the 322 mutations, there were 2 missense, 3 splice, 271 introns, 19 5'Flank, 4 3'UTR, 19 3'Flank, and 4 silent. Scores based on SIFT and PolyPhen-2 were used to rank the functional impact of mutations [3]. Within the 2 missense mutations, the breakdown was 1 deleterious and possibly damaging and 1 tolerated low confidence and benign. MutationMapper failed to annotate 525 mutations.

MB: Codes for a protein that binds to oxygen and nitric oxide, which helps alleviate any damage sustained from lack of oxygen to the heart [28]. Lack of MB is associated with CVDs, especially buildup of lipids in the heart [28]. To analyze the MB gene, we generated a lollipop plot of the MB sequence data on cBioPortal's MutationMapper software [2]. We found a total of 190 mutations mapped onto the MB sequence. Of the 190 mutations, there were 95 introns, 1 5'UTR, 39 5'Flank, 4 3'UTR, 49 3'Flank, and 2 silent. No functional impact scores were retrieved for any of these mutations. MutationMapper failed to annotate 738 mutations.

PDPN: Codes for a protein that plays a role in organ development including the heart and high amounts of PDPN are associated with CVDs, including heart attacks [29, 30]. To analyze the PDPN gene, we generated a lollipop plot of the PDPN sequence data on cBioPortal's MutationMapper software [2]. We found a total of 263 mutations mapped onto the PDPN sequence. Of the 263 mutations, there were 1 missense, 203 introns, 1 5'UTR, 35 5'Flank, 20 3'UTR, 1 3'Flank, and 2 silent. Scores based on SIFT and PolyPhen-2 were used to rank the functional impact of mutations [3]. The 1 missense generated was labeled as tolerated and benign. MutationMapper failed to annotate 351 mutations.

SLC2A1: A glucose transporter protein type 1 or GLUT1 protein which helps carry glucose into cells to use for energy and is important for brain function [31, 32]. GLUT1 deficiency syndrome is associated with symptoms of early infantile seizers, development delay, and transient movement disorders [31]. Irregular expression of SLC2A1 is also linked to CVDs [33].

To analyze the SLC2A1 gene, we generated a lollipop plot of the SLC2A1 sequence data on cBioPortal's MutationMapper software [2]. We found a total of 261 mutations mapped onto the SLC2A1 sequence. Of the 261 mutations, there were 1 splice, 216 introns, 2 5'UTR, 5 3'UTR, 30 3'Flank, and 7 silent. No functional impact scores were retrieved for any of these mutations. MutationMapper failed to annotate 230 mutations.

TAC1: A gene that produces a protein known as substance P and higher levels of substance P are linked to inflammation of the heart and other [34]. To analyze the TAC1 gene, we generated a lollipop plot of the TAC1 sequence data on cBioPortal's MutationMapper software [2]. We found a total of 103 mutations mapped onto the TAC1 sequence. Of the 103 mutations, there were 36 introns, 24 5'Flank, 5 3'UTR, and 38 3'Flank. No functional impact scores were retrieved for any of these mutations. MutationMapper failed to annotate 2518 mutations.

TEK: Involved in many functions including influencing the growth of blood vessels and acts as a receptor protein for Angiopioetin-1; mutations in TEK are associated with irregular formation of blood vessels and

the heart and are linked to CVDs [35, 36]. To analyze the TEK gene, we generated a lollipop plot of the TEK sequence data on cBioPortal's MutationMapper software [2]. We found a total of 1261 mutations mapped onto the TEK sequence. Of the 1261 mutations, there were 8 missense, 1 splice, 1120 introns, 46 5'Flank, 8 3'UTR, 69 3'Flank, and 9 silent. Scores based on SIFT and PolyPhen-2 were used to rank the functional impact of mutations [3]. Among the 8 missense mutations generated, the breakdown was 5 tolerated and benign, 1 tolerated and probably damaging, 1 deleterious and possibly damaging, and 1 deleterious and benign. MutationMapper failed to annotate 518 mutations.

TRPV1: Codes for a protein channel whose activity is catalyzed by temperature and pH changes [37]. TRPV1 activity has been linked to high blood pressure, thickhening of the heart, and other CVDs [38]. To analyze the TRPV1 gene, we generated a lollipop plot of the TRPV1 sequence data on cBioPortal's MutationMapper software [2]. We found a total of 337 mutations mapped onto the TRPV1 sequence. Of the 337 mutations, there were 8 missense, 1 truncating, 207 introns, 3 5'UTR, 68 5'Flank, 7 3'UTR, 38 3'Flank, and 5 silent. Scores based on SIFT and PolyPhen-2 were used to rank the functional impact of mutations [3]. Among the 8 missense mutations generated, the breakdown was 7 tolerated and benign and 1 deleterious and benign. MutationMapper failed to annotate 256 mutations.

SMUG1: A protein that assists in removing uracil from single-stranded and double-stranded DNA during the process of base excision repair. Studies have linked breast cancer and CVDs with the downregulation of SMUG1 [39]. To analyze the SMUG1 gene, we generated a lollipop plot of the SMUG1 sequence data on cBioPortal's MutationMapper software [2]. We found a total of 112 mutations mapped onto the SMUG1 sequence. Of the 112 mutations, there were 1 missense, 1 truncating, 1 splice, 61 introns, 1 5'UTR, 17 5'Flank, 4 3'UTR, and 26 3'Flank. The 1 missense mutation generated was classified as tolerated low confidence and benign. MutationMapper failed to annotate 433 mutations.

ZBTB8OS: A gene associated with tRNA splicing which helps to enable metal during metal ion binding activity [40]. ZBTB8OS can regulate levels of XBP1, which has been linked to numerous CVDs and cancers including B-cell leukemias [40]. To analyze the ZBTB8OS gene, we generated a lollipop plot of the ZBTB8OS sequence data on cBioPortal's MutationMapper software [2]. We found a total of 233 mutations mapped onto the ZBTB8OS sequence. Of the 233 mutations, there were 197 introns, 12 3'UTR, 23 3'Flank, and 1 silent. No functional impact scores were retrieved for any of these mutations. MutationMapper failed to annotate 205 mutations.

References

1. Lipskaia, L., Chemaly, E. R., Hadri, L., Lompre, A. M., & Hajjar, R. J. (2010). Sarcoplasmic reticulum Ca(2+) ATPase as a therapeutic target for heart failure. Expert opinion on biological therapy, 10(1), 29–41. https://doi.org/10.1517/14712590903321462

2. Vohra, S., & Biggin, P. C. (2013). Mutationmapper: a tool to aid the mapping of protein mutation data. *PloS one*, *8*(8), e71711. https://doi.org/10.1371/journal.pone.0071711

3. Flanagan, S. E., Patch, A. M., & Ellard, S. (2010). Using SIFT and PolyPhen to predict loss-of-function and gain-of-function mutations. *Genetic testing and molecular biomarkers*, *14*(4), 533–537. https://doi.org/10.1089/gtmb.2010.0036

4. Ng, P. C., & Henikoff, S. (2003). SIFT: Predicting amino acid changes that affect protein function. Nucleic acids research, 31(13), 3812–3814.

5. Sim, N. L., Kumar, P., Hu, J., Henikoff, S., Schneider, G., & Ng, P. C. (2012). SIFT web server: predicting effects of amino acid substitutions on proteins. Nucleic acids research, 40(Web Server issue), W452–W457.

6. Kumar, P., Henikoff, S., & Ng, P. C. (2009). Predicting the effects of coding non-synonymous variants on protein function using the SIFT algorithm. Nature protocols, 4(7), 1073–1081.

7. Adzhubei, I., Jordan, D. M., & Sunyaev, S. R. (2013). Predicting functional effect of human missense mutations using PolyPhen-2. Current protocols in human genetics, Chapter 7, Unit7.20.

8. Hai C. M. (2008). Caldesmon as a therapeutic target for proliferative vascular diseases. Mini reviews in medicinal chemistry, 8(12), 1209–1213. https://doi.org/10.2174/138955708786140981

9. Zheng, P. P., Severijnen, L. A., van der Weiden, M., Willemsen, R., & Kros, J. M. (2009). A crucial role of caldesmon in vascular development in vivo. Cardiovascular research, 81(2), 362–369. https://doi.org/10.1093/cvr/cvn294

10. Mackie, A. R., & Losordo, D. W. (2011). CD34-positive stem cells: in the treatment of heart and vascular disease in human beings. Texas Heart Institute journal, 38(5), 474–485.

11. Daub, S., Lutgens, E., Münzel, T., & Daiber, A. (2020). CD40/CD40L and Related Signaling Pathways in Cardiovascular Health and Disease-The Pros and Cons for Cardioprotection. International journal of molecular sciences, 21(22), 8533. https://doi.org/10.3390/ijms21228533

12. Jiang, Y., Zhu, Y., Qiu, W., Liu, Y. J., Cheng, G., Liu, Z. J., & Ouyang, S. (2017). Structural and functional analyses of human DDX41 DEAD domain. Protein & cell, 8(1), 72–76. https://doi.org/10.1007/s13238-016-0351-9

13. Bannon, S. A., Routbort, M. J., Montalban-Bravo, G., Mehta, R. S., Jelloul, F. Z., Takahashi, K., Daver, N., Oran, B., Pemmaraju, N., Borthakur, G., Naqvi, K., Issa, G., Sasaki, K., Alvarado, Y., Kadia, T. M., Konopleva, M., Shamanna, R. K., Khoury, J. D., Ravandi, F., Champlin, R., … DiNardo, C. D. (2021). Next-Generation Sequencing of DDX41 in Myeloid Neoplasms Leads to Increased Detection of Germline Alterations. Frontiers in oncology, 10, 582213. https://doi.org/10.3389/fonc.2020.582213

14. Chellapandian, D., Pole, J. D., Nathan, P. C., & Sung, L. (2019). Congestive heart failure among children with acute leukemia: a population-based matched cohort study. Leukemia & lymphoma, 60(2), 385–394. https://doi.org/10.1080/10428194.2018.1474522

15. Vizin, T., & Kos, J. (2015). Gamma-enolase: a well-known tumour marker, with a less-known role in cancer. Radiology and oncology, 49(3), 217–226. https://doi.org/10.1515/raon-2015-0035

16. Streitberger, K. J., Leithner, C., Wattenberg, M., Tonner, P. H., Hasslacher, J., Joannidis, M., Pellis, T., Di Luca, E., Födisch, M., Krannich, A., Ploner, C. J., & Storm, C. (2017). Neuron-Specific Enolase Predicts Poor Outcome After Cardiac Arrest and Targeted Temperature Management: A Multicenter Study on 1,053 Patients. Critical care medicine, 45(7), 1145–1151. https://doi.org/10.1097/CCM.0000000000002335

17. Fan, Q., Huang, Z. M., Boucher, M., Shang, X., Zuo, L., Brinks, H., Lau, W. B., Zhang, J., Chuprun, J. K., & Gao, E. (2013). Inhibition of Fas-associated death domain-containing protein (FADD) protects against myocardial ischemia/reperfusion injury in a heart failure mouse model. PloS one, 8(9), e73537. https://doi.org/10.1371/journal.pone.0073537

18. Yun, Y. R., Won, J. E., Jeon, E., Lee, S., Kang, W., Jo, H., Jang, J. H., Shin, U. S., & Kim, H. W. (2010). Fibroblast growth factors: biology, function, and application for tissue regeneration. Journal of tissue engineering, 2010, 218142. https://doi.org/10.4061/2010/218142

19. de Wit, M. C., de Coo, I. F., Lequin, M. H., Halley, D. J., Roos-Hesselink, J. W., & Mancini, G. M. (2011). Combined cardiological and neurological abnormalities due to filamin A gene mutation. Clinical research in cardiology : official journal of the German Cardiac Society, 100(1), 45–50. https://doi.org/10.1007/s00392-010-0206-y

20. Zaidieh, T., Habbal, W., & Monem, F. (2015). Screening of GJB6 gene large deletions among Syrians with congenital hearing impairment. Genetic testing and molecular biomarkers, 19(7), 405–407. https://doi.org/10.1089/gtmb.2015.0019

21. Yang, R., Hu, Z., Kong, Q., Li, W., Zhang, L., Du, X., Huang, S., Xia, X., & Sang, H. (2016). A known mutation in GJB6 in a large Chinese family with hidrotic ectodermal dysplasia. Journal of the European Academy of Dermatology and Venereology : JEADV, 30(8), 1362–1365. https://doi.org/10.1111/jdv.13600

22. Brouillard, P., Boon, L. M., Mulliken, J. B., Enjolras, O., Ghassibé, M., Warman, M. L., Tan, O. T., Olsen, B. R., & Vikkula, M. (2002). Mutations in a novel factor, glomulin, are responsible for glomuvenous malformations ("glomangiomas"). American journal of human genetics, 70(4), 866–874. https://doi.org/10.1086/339492

23. Ahmed, M. H., Ghatge, M. S., & Safo, M. K. (2020). Hemoglobin: Structure, Function and Allostery. Sub-cellular biochemistry, 94, 345–382. https://doi.org/10.1007/978-3-030-41769-7_14

24. Chonchol, M., & Nielson, C. (2008). Hemoglobin levels and coronary artery disease. American heart journal, 155(3), 494–498. https://doi.org/10.1016/j.ahj.2007.10.031

25. Sauvageau, M., Goff, L. A., Lodato, S., Bonev, B., Groff, A. F., Gerhardinger, C., Sanchez-Gomez, D. B., Hacisuleyman, E., Li, E., Spence, M., Liapis, S. C., Mallard, W., Morse, M., Swerdel, M. R., D'Ecclessis, M. F., Moore, J. C., Lai, V., Gong, G., Yancopoulos, G. D., Frendewey, D., … Rinn, J. L. (2013). Multiple knockout mouse models reveal lincRNAs are required for life and brain development. eLife, 2, e01749. https://doi.org/10.7554/eLife.01749

26. Lin, F., Morrison, J. M., Wu, W., & Worman, H. J. (2005). MAN1, an integral protein of the inner nuclear membrane, binds Smad2 and Smad3 and antagonizes transforming growth factor-beta signaling. Human molecular genetics, 14(3), 437–445. https://doi.org/10.1093/hmg/ddi040

27. Mumm, S., Wenkert, D., Zhang, X., McAlister, W. H., Mier, R. J., & Whyte, M. P. (2007). Deactivating germline mutations in LEMD3 cause osteopoikilosis and Buschke-Ollendorff syndrome, but not sporadic melorheostosis. Journal of bone and mineral research : the official journal of the American Society for Bone and Mineral Research, 22(2), 243–250. https://doi.org/10.1359/jbmr.061102

28. Hendgen-Cotta, U. B., Esfeld, S., Coman, C., Ahrends, R., Klein-Hitpass, L., Flögel, U., Rassaf, T., & Totzeck, M. (2017). A novel physiological role for cardiac myoglobin in lipid metabolism. Scientific reports, 7, 43219. https://doi.org/10.1038/srep43219

29. Astarita, J. L., Acton, S. E., & Turley, S. J. (2012). Podoplanin: emerging functions in development, the immune system, and cancer. Frontiers in immunology, 3, 283. https://doi.org/10.3389/fimmu.2012.00283

30. Cimini, M., Garikipati, V., de Lucia, C., Cheng, Z., Wang, C., Truongcao, M. M., Lucchese, A. M., Roy, R., Benedict, C., Goukassian, D. A., Koch, W. J., & Kishore, R. (2019). Podoplanin neutralization improves cardiac remodeling and function after acute myocardial infarction. JCI insight, 5(15), e126967. https://doi.org/10.1172/jci.insight.126967

31. Wang, D., Kranz-Eble, P., & De Vivo, D. C. (2000). Mutational analysis of GLUT1 (SLC2A1) in Glut-1 deficiency syndrome. Human mutation, 16(3), 224–231. https://doi.org/10.1002/1098-1004(200009)16:3<224::AID-HUMU5>3.0.CO;2-P

32. Koch, H., & Weber, Y. G. (2019). The glucose transporter type 1 (Glut1) syndromes. *Epilepsy & behavior : E&B*, *91*, 90–93. https://doi.org/10.1016/j.yebeh.2018.06.010

33. Szablewski L. (2017). Glucose transporters in healthy heart and in cardiac disease. International journal of cardiology, 230, 70–75. https://doi.org/10.1016/j.ijcard.2016.12.083

34. Dehlin, H. M., & Levick, S. P. (2014). Substance P in heart failure: the good and the bad. International journal of cardiology, 170(3), 270–277. https://doi.org/10.1016/j.ijcard.2013.11.010

35. Limaye, N., Wouters, V., Uebelhoer, M., Tuominen, M., Wirkkala, R., Mulliken, J. B., Eklund, L., Boon, L. M., & Vikkula, M. (2009). Somatic mutations in angiopoietin receptor gene TEK cause solitary and multiple sporadic venous malformations. Nature genetics, 41(1), 118–124. https://doi.org/10.1038/ng.272

36. Eklund, L., Kangas, J., & Saharinen, P. (2017). Angiopoietin-Tie signalling in the cardiovascular and lymphatic systems. Clinical science (London, England : 1979), 131(1), 87–103. https://doi.org/10.1042/CS20160129

37. Bonvini, S. J., Birrell, M. A., Grace, M. S., Maher, S. A., Adcock, J. J., Wortley, M. A., Dubuis, E., Ching, Y. M., Ford, A. P., Shala, F., Miralpeix, M., Tarrason, G., Smith, J. A., & Belvisi, M. G. (2016). Transient receptor potential cation channel, subfamily V, member 4 and airway sensory afferent activation: Role of adenosine triphosphate. The Journal of allergy and clinical immunology, 138(1), 249–261.e12. https://doi.org/10.1016/j.jaci.2015.10.044

38. Buckley, C. L., & Stokes, A. J. (2011). Mice lacking functional TRPV1 are protected from pressure overload cardiac hypertrophy. Channels (Austin, Tex.), 5(4), 367–374. https://doi.org/10.4161/chan.5.4.17083

39. Abdel-Fatah, T. M., Albarakati, N., Bowell, L., Agarwal, D., Moseley, P., Hawkes, C., Ball, G., Chan, S., Ellis, I. O., & Madhusudan, S. (2013). Single-strand selective monofunctional uracil-DNA glycosylase (SMUG1) deficiency is linked to aggressive breast cancer and predicts response to adjuvant therapy. Breast cancer research and treatment, 142(3), 515–527. https://doi.org/10.1007/s10549-013-2769-6

40. Jurkin, J., Henkel, T., Nielsen, A. F., Minnich, M., Popow, J., Kaufmann, T., Heindl, K., Hoffmann, T., Busslinger, M., & Martinez, J. (2014). The mammalian tRNA ligase complex mediates splicing of XBP1 mRNA and controls antibody secretion in plasma cells. The EMBO journal, 33(24), 2922–2936. https://doi.org/10.15252/embj.201490332
